# SARS-CoV-2 setting-specific transmission rates: a systematic review and meta-analysis

**DOI:** 10.1093/cid/ciab100

**Published:** 2021-02-09

**Authors:** Hayley A Thompson, Andria Mousa, Amy Dighe, Han Fu, Alberto Arnedo-Pena, Peter Barrett, Juan Bellido-Blasco, Qifang Bi, Antonio Caputi, Liling Chaw, Luigi De Maria, Matthias Hoffmann, Kiran Mahapure, Kangqi Ng, Jagadesan Raghuram, Gurpreet Singh, Biju Soman, Vicente Soriano, Francesca Valent, Luigi Vimercati, Liang En Wee, Justin Wong, Azra C Ghani, Neil M Ferguson

**Affiliations:** 1 MRC Centre for Global Infectious Disease Analysis & WHO Collaborating Centre for Infectious Disease Modelling, Abdul Latif Jameel Institute for Disease and Emergency Analytics, Imperial College London, London, UK; 2 Sección de Epidemiología. Centro de Salud Pública de Castellón, Valencia, Spain; 3 Centro de Investigación Biomédica en Red de Epidemiología y Salud Pública (CIBERESP), Valencia, Spain; 4 School of Public Health, University College Cork, Cork, Ireland; 5 Irish Centre for Maternal and Child Health Research (INFANT), University College Cork, Cork, Ireland; 6 Facultad de Ciencias de la Salud, Universitat Jaime I (UJI), Castelló, Spain; 7 Department of Epidemiology, Johns Hopkins Bloomberg School of Public Health, Baltimore, MD, USA; 8 Interdisciplinary Department of Medicine, University of Bari, Unit of Occupational Medicine, University Hospital of Bari, Bari, Italy; 9 PAPRSB Institute of Health Sciences, Universiti Brunei Darussalam, Jalan Tungku Link, Brunei; 10 Division of General Internal Medicine, Infectious Diseases and Hospital Epidemiology, Cantonal Hospital Olten, Switzerland; 11 Department of Plastic Surgery, Dr Prabhakar Kore Hospital and MRC, Belgaum, Karnataka, India; 12 Changi General Hospital, Singapore; 13 Sree Chitra Tirunal Institute for Medical Sciences and Technology, Thiruvananthapuram, Kerala, India; 14 UNIR Health Sciences School & Medical Center, Madrid, Spain; 15 SOC Istituto di Igiene ed Epidemiologia Clinica, Azienda Sanitaria Universitaria Friuli Centrale, Udine, Italy; 16 Department of Infectious Diseases, Singapore General Hospital, Singapore, Singapore; 17 Disease Control Division, Ministry of Health, Brunei

**Keywords:** SARS-CoV-2, COVID-19, transmission, secondary attack rate, contact tracing

## Abstract

**Background:**

Understanding the drivers of SARS-CoV-2 transmission is crucial for control policies but evidence of transmission rates in different settings remains limited.

**Methods:**

We conducted a systematic review to estimate secondary attack rates (SAR) and observed reproduction numbers (R_obs_) in different settings exploring differences by age, symptom status, and duration of exposure. To account for additional study heterogeneity, we employed a Beta-Binomial model to pool SARs across studies and a Negative-binomial model to estimate R_obs_.

**Results:**

Households showed the highest transmission rates, with a pooled SAR of 21.1% (95%CI:17.4%-24.8%). SARs were significantly higher where the duration of household exposure exceeded 5 days compared with exposure of ≤5 days. SARs related to contacts at social events with family and friends were higher than those for low-risk casual contacts (5.9% vs. 1.2%). Estimates of SAR and R_obs_ for asymptomatic index cases were approximately a seventh, and for pre-symptomatic two thirds of those for symptomatic index cases. We found some evidence for reduced transmission potential both from and to individuals under 20 years of age in the household context, which is more limited when examining all settings.

**Conclusions:**

Our results suggest that exposure in settings with familiar contacts increases SARS-CoV-2 transmission potential. Additionally, the differences observed in transmissibility by index case symptom status and duration of exposure have important implications for control strategies such as contact tracing, testing and rapid isolation of cases. There was limited data to explore transmission patterns in workplaces, schools, and care-homes, highlighting the need for further research in such settings.

### Introduction

The SARS-CoV-2 virus which emerged in China in late 2019 has since spread rapidly around the world, with over 90 million confirmed cases and over 2 million deaths reported globally by January 2021 [1]. The severity of the infection, particularly in the oldest age-groups [2,3], has resulted in many countries implementing socially disruptive interventions to prevent onward spread. Early interventions focused on case isolation alongside identification of close contacts. In countries where these measures were insufficient to contain the virus, other non-pharmaceutical interventions (NPI) were introduced, including “stay-at-home” recommendations, closing schools, working from home and wearing face-masks. Governments are continually faced with the challenge of balancing social and economic harms caused by these NPI against the resurgence of cases. It is therefore critical to improve understanding of where transmission is taking place so that public health interventions can be better targeted.

To date, there have been relatively few detailed systematic epidemiological studies on transmission of the SARS-CoV-2 virus, with most published studies from the early epidemic in China and reviews focussing on household transmission [4,5]. These studies provide valuable information on key epidemiological statistics: the secondary attack rate (SAR), defined as the probability of onward infection from an index case among a defined group of close contacts and the observed reproduction number ($R_{obs}$), defined as the observed average number of secondary cases per index case. Quantifying these parameters can help us understand the relative role that different settings play in sustaining transmission through identifying the location and types of contacts that constitute higher transmission potential.

Here we present a systematic review to estimate SAR and $R_{obs}$ of SARS-CoV-2 in households, schools, workplaces, healthcare facilities and social settings. In addition, we examine differences in these parameters by age of index cases and their contacts, duration of household exposure to the index case, household size and symptom status of index cases.

### Methods

#### Systematic Review

##### *Data Source and Search Strategy*

We searched MEDLINE, Embase, MedRxiv, BioRxiv, arXiv and Wellcome Open Research with no language restrictions up to July 6$^{th}$ 2020, using the search strategy: ("COVID-19"OR"Coronavirus"OR "SARS-CoV-2"OR"2019-nCoV")AND("attack rate*"OR"contact*"OR"cluster*"), adapted for the preprint servers by removing Boolean operators and testing all possible search-term combinations. Studies were screened according to titles and abstracts, and then by review of full-texts and their bibliographies. Two reviewers (HAT and AM) screened the studies independently, using predetermined criteria. Differences were resolved through consensus and discussion with a third reviewer (AD). The study protocol can be accessed through PROSPERO (registration number: CRD42020200177).

##### *Inclusion criteria*

Eligible studies for review met the following criteria: 1) provided a definition of the case-contact setting, and 2) reported the number of index cases (defined as the first identified case), the number of secondary cases and the total number of contacts or a SAR and total number of contacts. Studies were included in the meta-analysis if they met two additional criteria 1) tested all contacts for SARS-CoV-2 infection regardless of symptom status and 2) reported on over one index case (to minimise publication bias in single case studies towards reporting larger outbreaks).

***Data Extraction***

We extracted summary data on study design, contact definition, testing method (RT-PCR/serology), testing strategy (all contacts irrespective of symptoms, symptomatic contacts only or a subset of contacts), and the number of index cases, contacts identified, contacts tested and secondary cases. Where available, we additionally extracted the following: age of index case, age of contacts, household size, duration of household exposure to an index case and symptom status of the index case. Data were obtained directly from the reports, but when not explicitly stated, we obtained additional data from study authors. Studies were assessed for risk of bias using a critical appraisal tool checklist for prevalence studies, adapted to this study [6]. Articles were given a quality score to reflect methodological rigour, clarity and transparency in reporting relevant to this study's outcomes.

**Statistical Analysis**

Articles eligible for meta-analysis were stratified into the following settings: households, schools, workplaces, healthcare, group-living, and social contacts. Within household contacts we undertook a subgroup analysis stratifying by household size and the duration between symptom onset/confirmation and isolation or hospitalisation of the index case. We explored age-dependent differences in infectivity and susceptibility across all contact locations and household contacts (stratified by ages 0-19 years and 20 years-plus). Finally, we examined differences in transmissibility by symptom status of index cases across all exposure locations due to limited studies stratifying by symptom status and exposure location. Stratifications were chosen to maximise the available data across studies.

Due to potential within-study correlation (e.g. individuals in the same locations experiencing the same public health interventions and country-specific home, travel and work practices) and between-study heterogeneity due to study and population differences, we employed a Beta-

Binomial model to pool SARs and a Poisson-Gamma mixture model to pool $R_{obs}$ across studies (Supplementary Methods)[7–9].

### Results

We identified 1,872 published studies, 78 of which were included after full-text screening (Figure 1). A further 22 eligible studies were identified through pre-print servers and bibliography screening. Of these 97 studies, summarised in Supplementary Tables 1-7, 67 tested all contacts regardless of symptom status. Among those, 45 reported data from >1 index case and were included in the meta-analysis.

#### Household

We identified 29 eligible studies reporting household contacts [10–38], with over half carried out in China (Supplementary Table 1). Household definitions were broadly consistent across studies, requiring contacts to be living in the same residence as the index case. One study additionally included non-resident contacts who spent ≤24 hours in the same residence as the index case [22](Supplementary Table 1), but its inclusion had no significant impact on the pooled SAR estimate.

Study estimates of household SARs ranged from 6.1%[25] to 51.2%[38] with a pooled estimate of 21.1% (95%CI:17.4%-24.8%)(Figure 2A). The relationship between the number of secondary cases and the number of index cases is shown in Supplementary Figure 1. $R_{obs}$ varied across studies from 0.05 [25] to 5.5 [38], with a pooled household $R_{obs}$ of 0.96 (95%CI:0.67-1.32)(Supplementary Table 8). The SAR increased with longer durations of exposure (14.2% [95%CI:5.8%-22.5%] with ≤5 days of exposure to an index case versus 34.9% [95%CI:16.3%-53.6%] with >5 days of exposure; p=0.05; Figure 2B). Longer durations of exposure were similarly associated with an increased pooled $R_{obs}$ (0.40 [95%CI:0.21-0.72] with ≤5 days to 1.91 [95%CI:0.86-3.55] with >5 days, p<0.001)(Supplementary Table 9). There was a trend for decreasing transmission with increasing household size, but this was not statistically significant (p=0.29)(Supplementary Figure 2). Study-level estimates for the small subset of studies reporting household sizes were highly variable.

### Workplaces

Seven studies reported workplace contacts (Supplementary Table 3)[13,27,28,31,39–41]. Three of these studies were cluster investigations from a single index case, including a boardroom meeting in Germany (SAR: 91.7% [95%CI:61.5%-99.8%, 11/12])[41], supermarket employees in China (SAR: 9.2% [95%CI:4.7%-15.8%, 10/120])[28] and call centre colleagues in South Korea (SAR: 43.5% [95%CI:36.8%-50.4%, 94/216])[40]. Excluding those, the pooled SAR reduced from 12.3% (95%CI:1.3%-22.5%)(Supplementary Figure 3) to 1.9% (95%CI:0.0%-3.9%)(Figure 3A). Workplace contacts likely represented a variety of sectors across studies, and only one study provided a detailed definition [40].

### Healthcare Facilities

Twenty-eight studies reported healthcare-based contacts, 10 of which met our inclusion criteria for meta-analysis (Supplementary Table 4)[30,33,39,42–46], including index case populations of patients [30,33,39,42,44,45], healthcare workers [43] or both [21,46]. Study-level SARs varied from 0.0%-17.1% resulting in a pooled estimate among any healthcare contacts of 3.6% (95%CI:1.0%-6.9%)(Figure 3B). There was no significant difference in the pooled SAR between patient or healthcare staff contact subgroups (p=0.64; Figure 3B). The estimated $R_{obs}$ in healthcare settings was 1.18 (95%CI:0.65-2.04) but ranged between 0.0 to 4.5 (Supplementary Table 10, Supplementary Figure 1).

### Social Settings

Twenty-two studies were identified with social contact settings including travelling, religious events, fitness classes, shopping, entertainment venues and events with family and friends (Supplementary Table 6). Of these, 13 fulfilled the criteria for meta-analysis [10,11,39,45,47,13,17,26–28,30,31,37]. We estimate low SARs in low-contact events with casual contacts or strangers, with a pooled SAR of 1.2% (95%CI:0.3%-2.1%)(Figure 3C). In contrast, in settings with more familiar and prolonged contact such as events with family and friends, the pooled SAR was 5.9% (95%CI:3.8%-8.1%) and $R_{obs}$ was 0.38 (95%CI:0.18-0.64)(Supplementary Table 11, Supplementary Figure 1). Travel-related contacts had an estimated SAR of 5.0% (95%CI:0.3%-9.8%), similar to that in social events with family and friends (Figure 3C, Supplementary Table 6). $R_{obs}$ could not be estimated for any other contact groups due to insufficient data. Several other social settings with high levels of transmission were identified but could not be pooled (Supplementary Results).

### Exposure Location Summary

Table 1 summarises the pooled estimates of SAR and $R_{obs}$ across different exposure locations. While the highest SARs were estimated for household contacts and in familial settings, the highest $R_{obs}$ was estimated for healthcare settings. Pooled estimates for schools and care-homes were not possible due to scarcity of studies (Supplementary Results).

### Age Effects

Ten studies provided age breakdowns of index cases and contacts [10,12,16,22,25,31,33,37,45,48] meeting the inclusion criteria. Pooling across all exposure locations we found no significant differences in transmissibility or susceptibility by age of index cases or contacts (Figure 4 and Supplementary Table 12, Supplementary Figures 4-5).

However, we observed weak evidence for a difference in transmissibility and a significant difference in susceptibility by age in household studies (index 0-19 years: 4.4% [95%CI:0.0%-10.8%] vs. index

≥20 years: 10.0% [95%CI:8.2%-11.8%], p=0.07 and contact 0-19 years: 6.16% [95%CI:4.1%-8.2%] vs. contact ≥20 years: 13.7% [95%CI:8.7%-18.7%], p=0.01)(Figure 4A, Figure 4C). When further disaggregating age groups, the trend for SARs to increase with increasing age of index cases and contacts was maintained (Figure 4B, Figure 4D).We note that across contact age groups the SARs increased at a higher rate for household contacts (Figure 4D).

#### Symptom Status of Index Case

From 18 studies [18,19,44,45,47,49–53,22,27,29–31,33,39,40] SAR was lowest for asymptomatic index cases at 1.9% (95% CI:0.5%-3.1%)(Figure 5)(asymptomatic definitions and follow-up durations for included studies shown in Supplementary Table 13). SARs were significantly higher for pre-symptomatic and symptomatic index cases, estimated at 9.3% (95%CI:4.5%-14.0%, p=0.01) and 13.6% (95%CI:9.7%-17.5%, p<0.001), respectively (Figure 5), with similar patterns observed for pooled $R_{obs}$ estimates. Asymptomatic index cases resulted in the lowest $R_{obs}$ of 0.17 (95%CI:0.04-0.45), followed by pre-symptomatic index cases ($R_{obs}$=0.78 [95%CI:0.36-1.44], p=0.004) and symptomatic index cases ($R_{obs}$=1.01 [95%CI:0.57-1.61], p<0.001)(Supplementary Table 14, Supplementary Figure 1).

### Discussion

This systematic review provides an indication of the types and places of contacts and index case/contact characteristics that facilitate SARS-CoV-2 transmission. We found evidence to suggest that more familiar prolonged contact increases the potential for transmission, as does the presence or potential for symptoms with moderate age-dependent effects observed at the household level. However, there was limited data to allow exploration of transmission patterns in workplaces, schools, and care-homes.

Sustained daily contact within households may explain the high SAR (21.1%) which is comparable to the pooled estimate of an earlier systematic review (18.1%)[5]. Our estimate hides substantial heterogeneity between studies, with reported SARs ranging between 0%-51%. This is similar to influenza household transmission, where SARs ranged from 1%-38% [54,55], but higher than those for SARS-1 (6-8%)[56,57]. We found evidence of reduced SARs and $R_{obs}$ when index cases were isolated within 5 days of symptom onset, suggesting that household transmission characteristics are influenced by contact tracing and isolation policies. While SARs could not be estimated in other residential settings, it is not unexpected that these locations report high study-specific attack rates. Elderly residents living in care homes are particularly vulnerable populations with high risk of severe outcomes and COVID-19-related mortality [2,3], and. Understanding dynamics of transmission in these environments is vital to prevent further outbreaks. With many countries continuing to recommend "stay-at-home" measures, with cases isolating inside households, it is likely that this location will continue to be important in sustaining transmission.

We estimated a relatively low SAR (3.6%) in healthcare settings, substantially lower than that observed during the early stages of the SARS-1 epidemic [58–60]. This is potentially driven by the large number of identified and tested contacts per index case and stricter measures such as mask mandates and austere visitor policies. Although the probability of onward infection per individual contact is low in healthcare settings, the high number of contacts can lead to more opportunities for infection, which may explain the higher $R_{obs}$ (1.18) in this setting. It is plausible that sufficient infection control and personal protective equipment (PPE) limited transmission potential in these settings but could not be explored due to insufficient data. Notably, all studies took place in high- and middle-income countries where PPE is likely more widely available which, in addition to the diversity of these healthcare systems, make generalisations about SARS-CoV-2 transmission rates in healthcare facilities difficult.

Despite a relatively low transmission potential in index cases who do not develop symptoms (SAR 1.9%), our findings suggest that SAR from exposure to pre-symptomatic index cases is similar to that of symptomatic cases (9.3% vs 13.6%, p=0.08). The difficulty in identifying pre-symptomatic cases, who have been estimated to account for 47% of onward transmission [61], is particularly challenging for control policies. Our results highlight the continued need for physical distancing policies and widescale testing and contact tracing to identify and isolate those cases not yet showing symptoms. With asymptomatic transmission rates estimated to be significantly lower, this could have positive implications for vaccines which protect against symptomatic disease, potentially reducing the risk of transmission also.

While studies reporting on the age breakdown of index cases and contacts were limited, we found evidence for age-dependence in transmissibility and susceptibility in the household context, which was more limited when pooling across all settings. There is significant uncertainty around these estimates due to the sparsity of data. Therefore, understanding potential age-dependent effects in transmissibility and susceptibility remains challenging. Observed age differences could relate to behavioural, contextual or biological factors which are not yet fully understood [62]. There is growing evidence to suggest that children tend to experience mild or asymptomatic infections [63–67], which may explain the potential differences observed in SAR from cases aged under 20 years old. Further to this, symptomatic testing policies may fail to detect childhood infections or identify children as index cases which could bias estimates.

In contrast to influenza in which schools are clearly important contributors to transmission [68,69], school-based studies on SARS-CoV-2 are limited due to reactive school closures. In addition to schools closing, many countries have and continue to encourage working-from-home practices. Furthermore, workplace settings vary greatly not just by country, but also between sectors, with some more able to facilitate Covid-19 safety measures, highlighting the difficulty in ascertaining a universal workplace SAR. Currently there is insufficient information to explore SARs in different types of schools, workplaces or sectors.

We estimate relatively low $R_{obs}$ across all settings, suggesting that the use of contact tracing and isolation activities in these studies is an effective measure at reducing onward transmission.

However, low numbers of studies were pooled and therefore there was considerable uncertainty in these estimates. Additionally, large cluster outbreaks can occur, as evidenced in several studies [28,31,38,40,41,47,70–73] and often, these events have the potential to overwhelm surveillance systems. The aggregate nature of the data used for our $R_{obs}$ estimates likely hides substantial individual-level heterogeneity in transmission potential, hindering estimation of the overdispersion factor relating to SARS-CoV-2 transmission. Characterising this potential for 'super-spreading-events' will be critical for further informing where to direct contact tracing efforts. Early evidence suggests that transmission is overdispersed with around 19% of cases resulting in 80% of transmission in Hong Kong [74] and modelling studies based on reported global cluster sizes which suggested that as few as 10% of cases could account for 80% of all SARS-CoV-2 transmission [75]. The large cluster outbreaks identified in this review tend to be reported in indoor social or workplace settings, potentially highlighting these locations as facilitating super-spreading-events [76].

There are several limitations to this study. As in any active outbreak response, data collected from multiple teams under different country guidelines are subject to variations in definitions, follow-up time and testing protocols, limiting interpretation of pooled data. Furthermore, individual studies suffer from reporting and recall bias in identifying all contacts of a confirmed case. Across settings, index cases were commonly enrolled after presenting for medical attention and therefore can be biased towards infections that result in symptomatic or more serious illness, potentially distorting transmission pathways [54]. Without phylogenetic sequencing it is difficult to confidently resolve these pathways which could bias SARs estimates, for example if the index case was asymptomatic and is instead identified as a secondary case of the symptomatic case that first presented for testing or if transmission occurs outside the household but is attributed to a household index case. Globally, there is still a lack of detailed data from contact tracing studies to fully explore differing transmission potential by more precise exposure locations or individual characteristics. In this meta-analysis the majority of studies came from China, where strict control policies were implemented, possibly limiting the generalisability of estimates. In addition these studies were performed prior to the identification of new virus variants with potentially enhanced transmission potential [77]. It will be important to contrast our estimates with studies distinguishing between variants when available. Finally, evidence is continually emerging from newspaper articles, government reports and press-conferences that report on large outbreaks in care-homes, schools, workplaces and hospitals. Such sources cannot be systematically searched or reliably cited, and this information is not translated into published contact tracing studies due to limited public health resources and the prioritisation of the ongoing emergency response.

In conclusion, early data suggest that SARS-CoV-2 transmission is highest in locations in which sustained and prolonged contacts are made, including households and other residential locations. With many countries continuing to issue stay-at-home measures, ensuring sufficient and early isolation of cases from other household members will be crucial to prevent spread. The identified similar transmission potential from pre-symptomatic and symptomatic cases is also challenging for control policies and highlights the importance of enhanced contact testing, that is not reliant on symptom status, rapid isolation, and physical distancing of both symptomatic and asymptomatic cases, as well as the continued use of appropriate PPE. Further research on transmission in different social settings, as well as in schools and workplaces, in which there are limited data to date, is required to continue to inform transmission reduction strategies.

### Notes

### Acknowledgements

We thank the following authors for providing information on primary studies included in this review and extracting additional relevant data: Dr Rachel Burke, Dr Hao-Yuan Cheng, Dr Tasuku Hara, Dr Hsien-Ho Lin, and all of their colleagues involved in the original publications. We would also like to acknowledge all members of the Imperial College COVID-19 Response Team.

### Disclaimer

The funders had no role in study design, data collection and analysis, decision to publish, or preparation of the manuscript. The views expressed are those of the authors and not necessarily those of the United Kingdom (UK) Department of Health and Social Care, the National Health Service, the National Institute for Health Research (NIHR), Public Health England (PHE), UK MRC, UKRI or European Union.

### Funding

This work was supported by the National Institute for Health Research Health Protection Research Unit (NIHR HPRU) in Modelling and Health Economics, a partnership between Public Health England, Imperial College London and the London School of Hygiene and Tropical Medicine (Grant number: NIHR200908). We acknowledge funding from the Medical Research Council (MRC) Centre for Global Infectious Disease Analysis (Grant number: MR/R015600/1), jointly funded by the UK Medical Research Council and the UK Foreign, Commonwealth & Development Office (FCDO), under the MRC/FCDO Concordat agreement and is also part of the EDCTP2 programme supported by the European Union. In addition, HAT and AM are supported by a UK Medical Research Council Studentship (Grant number: G98669).

### Conflicts of Interest

ACG reports grants from Medical Research Council during the conduct of the study; grants from Bill and Melinda Gates Foundation, Wellcome Trust, NIH, PATH, Medicines for Malaria Venture, and Integrated Vector Control Consortium and personal fees from Malaria programming from The Global Fund, outside the submitted work.

NMF reports serving as PI of a grant for the MRC Centre for Global Infection Disease Analysis (UK Medical Research Council), a grant funding the NIHR Health Protection Research Unit for Mathematical Modelling and Health Economics (UK National Institute of Health Research), and a grant funding the Jameel Institute of Disease and Emergency Analytics at Imperial College (Community Jameel), during the conduct of the study. NMF also reports serving as PI of a NIH U01 grant under the NIGMS MIDAS program (NIH NIGMS), as the Co-I on a grant modelling antivirals against dengue (Janssen Pharmaceuticals), as the PI of two grants from the Bill and Melinda Gates Foundation, including one funding the Vaccine Impact Modelling Consortium (Bill and Melinda Gates Foundation), and a grant from Gavi, funding the Vaccine Impact Modelling Consortium (Gavi, the Vaccine Alliance), outside the submitted work.

All other authors declare no conflicts of interest.

### Tables

**Table 1 Summary table of the pooled SAR and $R_{obs}$ for the exposure locations considered in this study.** Where values are missing there was not enough data available to estimate a pooled value. SAR: Secondary Attack Rate; $R_{obs}$: observed reproduction number

| Setting | Pooled SAR (%) | 95% Confidence Interval (%) | Pooled $R_{obs}$ | 95% Confidence Interval |
|---|---|---|---|---|
| Households | 21.1 | 17.4 - 24.8 | 0.96 | 0.67 - 1.32 |
| Social gatherings with family and friends | 5.9 | 3.8 - 8.1 | 0.38 | 0.18 - 0.64 |
| Travel | 5.0 | 0.3 - 9.8 | - | - |
| Healthcare | 3.6 | 1.0 - 6.9 | 1.18 | 0.65 - 2.04 |
| Workplace | 1.9 | 0.0 - 3.9 | - | - |
| Casual close contacts | 1.2 | 0.3 - 2.1 | - | - |

## Figure Legends

***Figure 1. PRISMA flow diagram of study selection***

***Figure 2. A) Pooled overall household Secondary Attack Rates.*** *Studies are ordered by the number of index cases reported in the study. Studies of large household contact tracing investigations were included irrespective of whether the number of index cases was reported in the study.* ***B) Stratified by duration of household exposure to symptomatic index case.*** *Studies are ordered by the number of index cases reported in the study as this was not given by exposure duration. Study level point estimates and binomial confidence intervals are shown along with the pooled beta-binomial summary across studies. Exposure duration to the index case before isolation or hospitalisation categories (≤5 days and >5 days) were selected to maximize usage of data.*

***Figure 3. Secondary attack rates stratified by exposure locations. A) Workplace based contacts. B) Healthcare based contacts****. Beta-binomial summary estimates are presented for patient and healthcare staff contacts of index-cases and a combined (non-disaggregated) contacts category. This 'combined contacts' summary estimate was pooled across all studies in the healthcare setting and includes two studies where disaggregated contact groups were not reported.* ***C) Social contact environments.*** *Studies are ordered by the number of index cases reported at the study level. Large population-level studies were included irrespective of whether the number of index cases was reported by the study.*

***Figure 4. Pooled estimates of Secondary Attack Rates by age of the index case and contacts stratified by contact location. (A)*** *Index cases stratified by 0-19 and 20+ age brackets and exposure location to the index case* ***(B)*** *index cases stratified by 10-,20- and 40-year age brackets.* ***C)*** *Contacts stratified by 0-19 and 20+ age brackets and exposure location* ***(D)*** *contacts stratified by 10, 20- and 40- year age brackets. Point estimates were obtained from fitting a beta-binomial model to pooled study data, with 95% Confidence Intervals shown by horizontal and vertical bars. All contacts combine studies regardless of exposure locations, and household only those studies relating to household transmission. The pooled household SAR for ages 60+ is not shown as there was insufficient data for this age group.*

***Figure 5. Estimated Secondary Attack Rates from asymptomatic, pre-symptomatic and symptomatic index cases.*** *Studies are ordered by the number of index cases reported in the study as shown in the figure. Pooled estimates combine all exposure locations listed. Combined exposure locations relate to contact tracing studies where close contacts were not disaggregated by exposure location. Asymptomatic index cases were defined as those with a positive SARS-CoV-2 RT-PCR test and no reported clinical symptoms up to discharge or end of follow-up (at least 14 days). Pre-symptomatic index cases were defined as those not reporting symptoms at the time of testing or during exposure but later developed symptoms. Symptomatic index cases reported COVID-19-associated symptoms at the time of sampling and/or during exposure.*

Figure 1

Figure 2

**A**

| Study | Country | Index cases | Secondary cases | Total contacts | Secondary Attack rate |
|---|---|---|---|---|---|
| Mahapure et al | India | 4 | 22 | 43 | 0.51 (95% CI: 0.35−0.67) |
| Burke et al | USA | 9 | 2 | 15 | 0.13 (95% CI: 0.02−0.40) |
| Zhang et al | China | 13 | 12 | 93 | 0.13 (95% CI: 0.07−0.21) |
| Bohmer et al | Germany | 16 | 5 | 24 | 0.21 (95% CI: 0.07−0.42) |
| Chaw et al | Brunei | 19 | 16 | 123 | 0.13 (95% CI: 0.08−0.20) |
| Wang et al | China | 25 | 10 | 43 | 0.23 (95% CI: 0.12−0.39) |
| Dawson et al | USA | 26 | 16 | 64 | 0.25 (95% CI: 0.15−0.37) |
| Dong et al | China | 26 | 53 | 259 | 0.20 (95% CI: 0.16−0.26) |
| Xin et al | China | 31 | 19 | 106 | 0.18 (95% CI: 0.11−0.27) |
| Wu et al | China | 35 | 48 | 148 | 0.32 (95% CI: 0.25−0.41) |
| Zhang et al | China | 38 | 10 | 62 | 0.16 (95% CI: 0.08−0.28) |
| van der Hoek et al | Netherlands | 54 | 49 | 174 | 0.28 (95% CI: 0.22−0.35) |
| Cheng et al | Taiwan | 100 | 10 | 151 | 0.07 (95% CI: 0.03−0.12) |
| Li et al | China | 105 | 64 | 392 | 0.16 (95% CI: 0.13−0.20) |
| Wang et al | China | 124 | 77 | 355 | 0.22 (95% CI: 0.18−0.26) |
| Zhang et al | China | 136 | 339 | 956 | 0.35 (95% CI: 0.32−0.39) |
| Wu et al | China | 144 | 50 | 280 | 0.18 (95% CI: 0.14−0.23) |
| Sun et al | China | 148 | 189 | 598 | 0.32 (95% CI: 0.28−0.35) |
| Rosenberg et al | USA | 155 | 131 | 343 | 0.38 (95% CI: 0.33−0.44) |
| Chen et al | China | 187 | 37 | 279 | 0.13 (95% CI: 0.10−0.18) |
| Jing et al | China | 215 | 103 | 784 | 0.13 (95% CI: 0.11−0.16) |
| Yung et al | Singapore | 223 | 13 | 213 | 0.06 (95% CI: 0.03−0.10) |
| Luo et al | China | 347 | 96 | 946 | 0.10 (95% CI: 0.08−0.12) |
| Bi et al | China | 391 | 77 | 686 | 0.11 (95% CI: 0.09−0.14) |
| Wang et al | China | 602 | 111 | 714 | 0.16 (95% CI: 0.13−0.18) |
| Dattner et al | Israel | 637 | 873 | 2716 | 0.32 (95% CI: 0.30−0.34) |
| Park et al | South Korea | 5706 | 1248 | 10592 | 0.12 (95% CI: 0.11−0.12) |
| Pollan et al | Spain | | 282 | 860 | 0.33 (95% CI: 0.30−0.36) |
| Yousaf et al | USA | | 47 | 195 | 0.24 (95% CI: 0.18−0.31) |
| **Beta−Binomial Summary** | | | | | **0.21 (95% CI: 0.17−0.25)** |
| γ = 0.054 | | | | | |

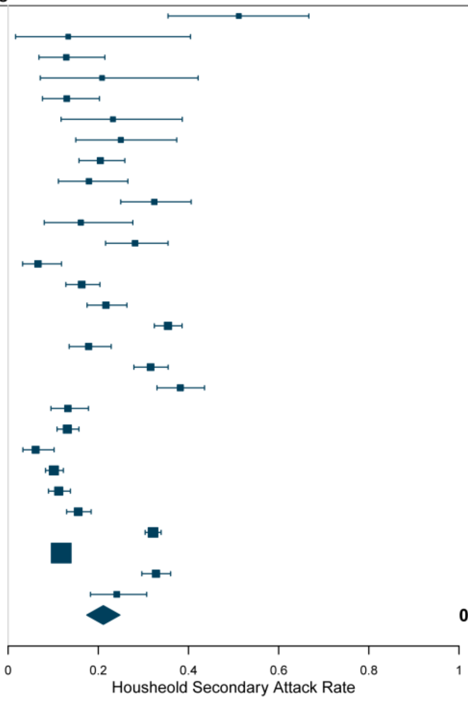

**B**

| Study | Country | Index cases | Secondary cases | Total contacts | Secondary Attack rate |
|---|---|---|---|---|---|
| **Up to 5 days exposure** | | | | | |
| Cheng et al | Taiwan | 100 | 3 | 77 | 0.04 (95% CI: 0.01−0.11) |
| Li et al | China | 105 | 24 | 139 | 0.17 (95% CI: 0.11−0.25) |
| Bohmer et al | Germany | 16 | 2 | 20 | 0.10 (95% CI: 0.01−0.32) |
| Chaw et al | Brunei | 19 | 14 | 109 | 0.13 (95% CI: 0.07−0.21) |
| Wu et al | China | 35 | 31 | 93 | 0.33 (95% CI: 0.24−0.44) |
| Burke et al | USA | 9 | 0 | 13 | 0.00 (95% CI: 0.00−0.25) |
| **Beta−Binomial Summary** | | | | | **0.14 (95% CI: 0.06−0.23)** |
| γ = 0.071 | | | | | |
| **Over 5 days exposure** | | | | | |
| Cheng et al | Taiwan | 100 | 7 | 74 | 0.09 (95% CI: 0.04−0.19) |
| Li et al | China | 105 | 40 | 253 | 0.16 (95% CI: 0.12−0.21) |
| Bohmer et al | Germany | 16 | 3 | 4 | 0.75 (95% CI: 0.19−0.99) |
| Chaw et al | Brunei | 19 | 2 | 14 | 0.14 (95% CI: 0.02−0.43) |
| Wu et al | China | 35 | 17 | 41 | 0.41 (95% CI: 0.26−0.58) |
| Mahapure et al | India | 4 | 22 | 43 | 0.51 (95% CI: 0.35−0.67) |
| Burke et al | USA | 9 | 2 | 2 | 1.00 (95% CI: 0.16−1.00) |
| **Beta−Binomial Summary** | | | | | **0.35 (95% CI: 0.16−0.54)** |
| γ = 0.166 | | | | | |

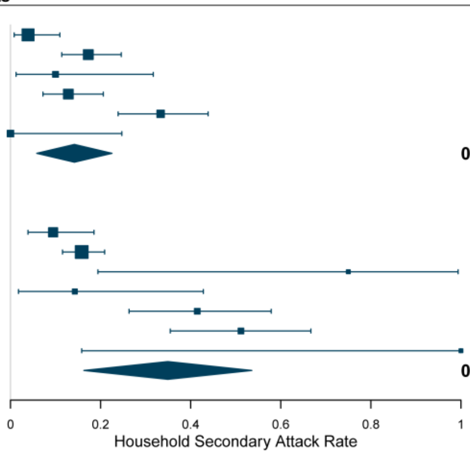

## Figure 3

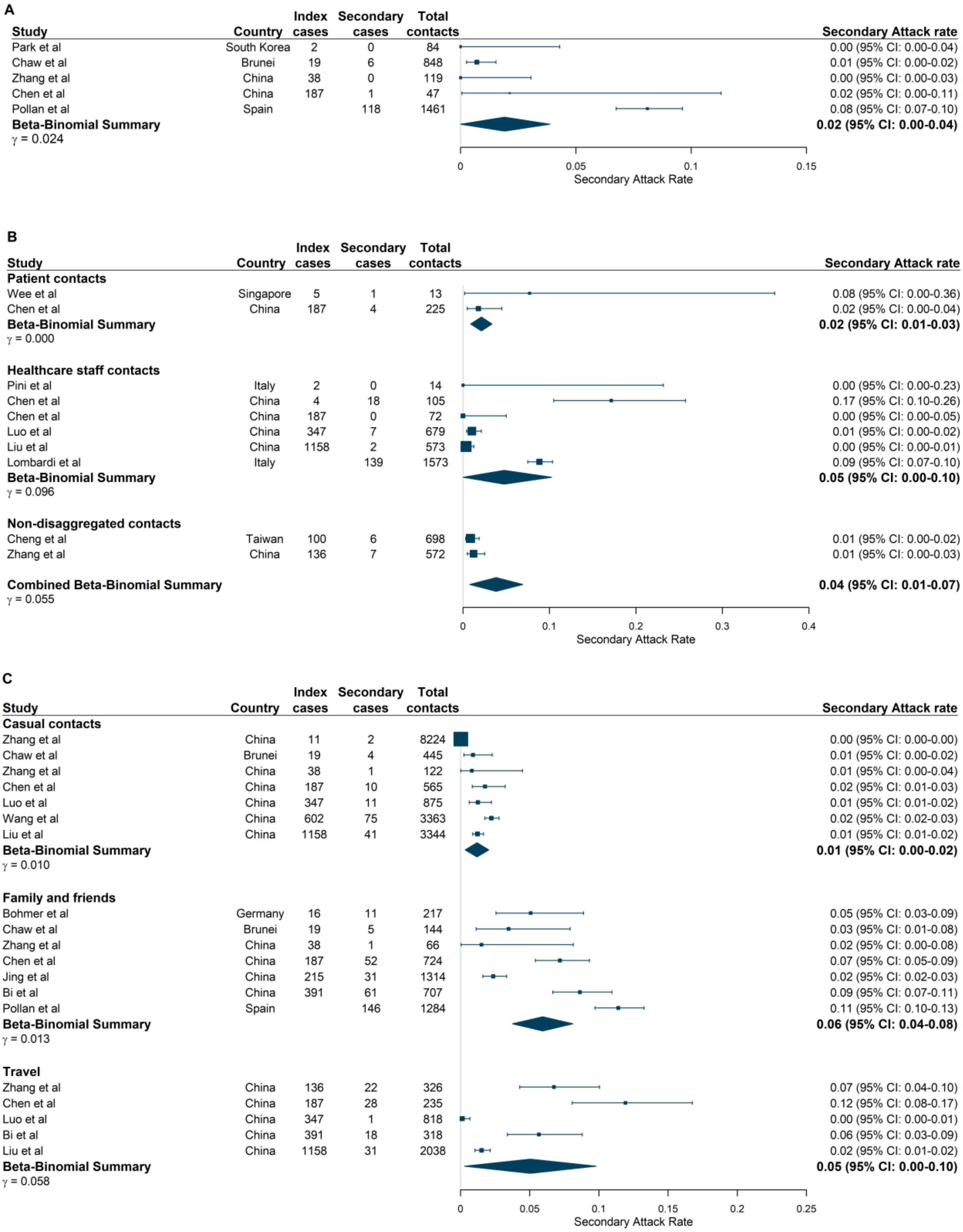

A
Study | Country | Index cases | Secondary cases | Total contacts | Secondary Attack rate
Park et al | South Korea | 2 | 0 | 84 | 0.00 (95% CI: 0.00-0.04)
Chaw et al | Brunei | 19 | 6 | 848 | 0.01 (95% CI: 0.00-0.02)
Zhang et al | China | 38 | 0 | 119 | 0.00 (95% CI: 0.00-0.03)
Chen et al | China | 187 | 1 | 47 | 0.02 (95% CI: 0.00-0.11)
Pollan et al | Spain | | 118 | 1461 | 0.08 (95% CI: 0.07-0.10)
Beta-Binomial Summary | 0.02 (95% CI: 0.00-0.04)
γ = 0.024
0
0.05
0.1
0.15
Secondary Attack Rate
B
Study | Country | Index cases | Secondary cases | Total contacts | Secondary Attack rate
Patient contacts
Wee et al | Singapore | 5 | 1 | 13 | 0.08 (95% CI: 0.00-0.36)
Chen et al | China | 187 | 4 | 225 | 0.02 (95% CI: 0.00-0.04)
Beta-Binomial Summary | 0.02 (95% CI: 0.01-0.03)
γ = 0.000
Healthcare staff contacts
Pini et al | Italy | 2 | 0 | 14 | 0.00 (95% CI: 0.00-0.23)
Chen et al | China | 4 | 18 | 105 | 0.17 (95% CI: 0.10-0.26)
Chen et al | China | 187 | 0 | 72 | 0.00 (95% CI: 0.00-0.05)
Luo et al | China | 347 | 7 | 679 | 0.01 (95% CI: 0.00-0.02)
Liu et al | China | 1158 | 2 | 573 | 0.00 (95% CI: 0.00-0.01)
Lombardi et al | Italy | | 139 | 1573 | 0.09 (95% CI: 0.07-0.10)
Beta-Binomial Summary | 0.05 (95% CI: 0.00-0.10)
γ = 0.096
Non-disaggregated contacts
Cheng et al | Taiwan | 100 | 6 | 698 | 0.01 (95% CI: 0.00-0.02)
Zhang et al | China | 136 | 7 | 572 | 0.01 (95% CI: 0.00-0.03)
Combined Beta-Binomial Summary | 0.04 (95% CI: 0.01-0.07)
γ = 0.055
0
0.1
0.2
0.3
0.4
Secondary Attack Rate
C
Study | Country | Index cases | Secondary cases | Total contacts | Secondary Attack rate
Casual contacts
Zhang et al | China | 11 | 2 | 8224 | 0.00 (95% CI: 0.00-0.00)
Chaw et al | Brunei | 19 | 4 | 445 | 0.01 (95% CI: 0.00-0.02)
Zhang et al | China | 38 | 1 | 122 | 0.01 (95% CI: 0.00-0.04)
Chen et al | China | 187 | 10 | 565 | 0.02 (95% CI: 0.01-0.03)
Luo et al | China | 347 | 11 | 875 | 0.01 (95% CI: 0.01-0.02)
Wang et al | China | 602 | 75 | 3363 | 0.02 (95% CI: 0.02-0.03)
Liu et al | China | 1158 | 41 | 3344 | 0.01 (95% CI: 0.01-0.02)
Beta-Binomial Summary | 0.01 (95% CI: 0.00-0.02)
γ = 0.010
Family and friends
Bohmer et al | Germany | 16 | 11 | 217 | 0.05 (95% CI: 0.03-0.09)
Chaw et al | Brunei | 19 | 5 | 144 | 0.03 (95% CI: 0.01-0.08)
Zhang et al | China | 38 | 1 | 66 | 0.02 (95% CI: 0.00-0.08)
Chen et al | China | 187 | 52 | 724 | 0.07 (95% CI: 0.05-0.09)
Jing et al | China | 215 | 31 | 1314 | 0.02 (95% CI: 0.02-0.03)
Bi et al | China | 391 | 61 | 707 | 0.09 (95% CI: 0.07-0.11)
Pollan et al | Spain | | 146 | 1284 | 0.11 (95% CI: 0.10-0.13)
Beta-Binomial Summary | 0.06 (95% CI: 0.04-0.08)
γ = 0.013
Travel
Zhang et al | China | 136 | 22 | 326 | 0.07 (95% CI: 0.04-0.10)
Chen et al | China | 187 | 28 | 235 | 0.12 (95% CI: 0.08-0.17)
Luo et al | China | 347 | 1 | 818 | 0.00 (95% CI: 0.00-0.01)
Bi et al | China | 391 | 18 | 318 | 0.06 (95% CI: 0.03-0.09)
Liu et al | China | 1158 | 31 | 2038 | 0.02 (95% CI: 0.01-0.02)
Beta-Binomial Summary | 0.05 (95% CI: 0.00-0.10)
γ = 0.058
0
0.05
0.1
0.15
0.2
0.25
Secondary Attack Rate

Figure 4

Figure 5

Accepted M
