# Severe Acute Respiratory Syndrome Coronavirus 2 (SARS-CoV-2) Setting-specific Transmission Rates: A Systematic Review and Meta-analysis

**DOI:** 10.1093/cid/ciab100

**Published:** 2021-02-09

**Authors:** Hayley A Thompson, Andria Mousa, Amy Dighe, Han Fu, Alberto Arnedo-Pena, Peter Barrett, Juan Bellido-Blasco, Qifang Bi, Antonio Caputi, Liling Chaw, Luigi De Maria, Matthias Hoffmann, Kiran Mahapure, Kangqi Ng, Jagadesan Raghuram, Gurpreet Singh, Biju Soman, Vicente Soriano, Francesca Valent, Luigi Vimercati, Liang En Wee, Justin Wong, Azra C Ghani, Neil M Ferguson

**Affiliations:** 1 MRC Centre for Global Infectious Disease Analysis & World Health Organization Collaborating Centre for Infectious Disease Modelling, Abdul Latif Jameel Institute for Disease and Emergency Analytics, Imperial College London, London, UK; 2 Sección de Epidemiología, Centro de Salud Pública de Castellón, Valencia, Spain; 3 Centro de Investigación Biomédica en Red de Epidemiología y Salud Pública (CIBERESP), Valencia, Spain; 4 School of Public Health, University College Cork, Cork, Ireland; 5 Irish Centre for Maternal and Child Health Research (INFANT), University College Cork, Cork, Ireland; 6 Facultad de Ciencias de la Salud, Universitat Jaime I (UJI), Castelló, Spain; 7 Department of Epidemiology, Johns Hopkins Bloomberg School of Public Health, Baltimore, Maryland, USA; 8 Interdisciplinary Department of Medicine, University of Bari, Unit of Occupational Medicine, University Hospital of Bari, Bari, Italy; 9 PAPRSB Institute of Health Sciences, Universiti Brunei Darussalam, Jalan Tungku Link, Brunei; 10 Division of General Internal Medicine, Infectious Diseases and Hospital Epidemiology, Cantonal Hospital Olten, Olten, Switzerland; 11 Department of Plastic Surgery, Dr Prabhakar Kore Hospital and MRC, Belgaum, Karnataka, India; 12 Changi General Hospital, Singapore; 13 Sree Chitra Tirunal Institute for Medical Sciences and Technology, Thiruvananthapuram, Kerala, India; 14 UNIR Health Sciences School & Medical Center, Madrid, Spain; 15 SOC Istituto di Igiene ed Epidemiologia Clinica, Azienda Sanitaria Universitaria Friuli Centrale, Udine, Italy; 16 Department of Infectious Diseases, Singapore General Hospital, Singapore, Singapore; 17 Disease Control Division, Ministry of Health, Brunei

**Keywords:** SARS-CoV-2, COVID-19, transmission, secondary attack rate, contact tracing

## Abstract

**Background:**

Understanding the drivers of severe acute respiratory syndrome coronavirus 2 (SARS-CoV-2) transmission is crucial for control policies, but evidence of transmission rates in different settings remains limited.

**Methods:**

We conducted a systematic review to estimate secondary attack rates (SARs) and observed reproduction numbers (R_obs_) in different settings exploring differences by age, symptom status, and duration of exposure. To account for additional study heterogeneity, we employed a beta-binomial model to pool SARs across studies and a negative-binomial model to estimate R_obs_.

**Results:**

Households showed the highest transmission rates, with a pooled SAR of 21.1% (95% confidence interval [CI]:17.4–24.8). SARs were significantly higher where the duration of household exposure exceeded 5 days compared with exposure of ≤5 days. SARs related to contacts at social events with family and friends were higher than those for low-risk casual contacts (5.9% vs 1.2%). Estimates of SARs and R_obs_ for asymptomatic index cases were approximately one-seventh, and for presymptomatic two-thirds of those for symptomatic index cases. We found some evidence for reduced transmission potential both from and to individuals younger than 20 years of age in the household context, which is more limited when examining all settings.

**Conclusions:**

Our results suggest that exposure in settings with familiar contacts increases SARS-CoV-2 transmission potential. Additionally, the differences observed in transmissibility by index case symptom status and duration of exposure have important implications for control strategies, such as contact tracing, testing, and rapid isolation of cases. There were limited data to explore transmission patterns in workplaces, schools, and care homes, highlighting the need for further research in such settings.

## Teaser

In this review, we systematically explore factors that affect transmission of SARS-CoV-2, such as age, symptom status, setting, and duration of contact. Our results highlight the importance of targeting interventions in settings where close and prolonged contacts occur.

The severe acute respiratory syndrome coronavirus 2 (SARS-CoV-2) virus that emerged in China in late 2019 has since spread rapidly around the world, with more than 90 million confirmed cases and more than 2 million deaths reported globally by January 2021 [1]. The severity of the infection, particularly in the oldest age groups [2, 3], has resulted in many countries implementing socially disruptive interventions to prevent onward spread. Early interventions focused on case isolation alongside identification of close contacts. In countries where these measures were insufficient to contain the virus, other nonpharmaceutical interventions (NPIs) were introduced, including “stay-at-home” recommendations, closing schools, working from home, and wearing face masks. Governments are continually faced with the challenge of balancing social and economic harms caused by these NPIs against the resurgence of cases. It is therefore critical to improve understanding of where transmission is taking place so that public health interventions can be better targeted.

To date, there have been relatively few detailed systematic epidemiological studies on transmission of the SARS-CoV-2 virus, with most published studies from the early epidemic in China and reviews focusing on household transmission [4, 5]. These studies provide valuable information on key epidemiological statistics: the secondary attack rate (SAR), defined as the probability of onward infection from an index case among a defined group of close contacts and the observed reproduction numbers (R_obs_), defined as the observed average number of secondary cases per index case. Quantifying these parameters can help us understand the relative role that different settings play in sustaining transmission through identifying the location and types of contacts that constitute higher transmission potential.

Here, we present a systematic review to estimate SAR and R_obs_ of SARS-CoV-2 in households, schools, workplaces, healthcare facilities, and social settings. In addition, we examine differences in these parameters by age of index cases and their contacts, duration of household exposure to the index case, household size, and symptom status of index cases.

## METHODS

### Systematic Review

#### Data Source and Search Strategy

We searched MEDLINE, Embase, MedRxiv, BioRxiv, arXiv, and Wellcome Open Research with no language restrictions up to July 6, 2020, using the search strategy: (“COVID-19” OR “Coronavirus” OR “SARS-CoV-2” OR “2019-nCoV”) AND (“attack rate*” OR “contact*” “OR “cluster*”), adapted for the preprint servers by removing Boolean operators and testing all possible search-term combinations. Studies were screened according to titles and abstracts, and then by review of full texts and their bibliographies. Two reviewers (H. A. T. and A. M.) screened the studies independently using predetermined criteria. Differences were resolved through consensus and discussion with a third reviewer (A. D.). The study protocol can be accessed through PROSPERO (registration number: CRD42020200177).

#### Inclusion Criteria

Eligible studies for review met the following criteria: (1) provided a definition of the case-contact setting and (2) reported the number of index cases (defined as the first identified case), the number of secondary cases and the total number of contacts, or a SAR and total number of contacts. Studies were included in the meta-analysis if they met 2 additional criteria (1) tested all contacts for SARS-CoV-2 infection regardless of symptom status and (2) reported on more than 1 index case (to minimize publication bias in single-case studies toward reporting larger outbreaks).

#### Data Extraction

We extracted summary data on study design, contact definition, testing method (reverse transcriptase-polymerase chain reaction/serology), testing strategy (all contacts irrespective of symptoms, symptomatic contacts only, or a subset of contacts), and the number of index cases, contacts identified, contacts tested, and secondary cases. Where available, we additionally extracted the following: age of index case, age of contacts, household size, duration of household exposure to an index case, and symptom status of the index case. Data were obtained directly from the reports, but when not explicitly stated, we obtained additional data from study authors. Studies were assessed for risk of bias using a critical appraisal tool checklist for prevalence studies, adapted to this study [6]. Articles were given a quality score to reflect methodological rigor, clarity, and transparency in reporting relevant to this study’s outcomes.

### Statistical Analysis

Articles eligible for meta-analysis were stratified into the following settings: households, schools, workplaces, healthcare, group living, and social contacts. Within household contacts, we undertook a subgroup analysis stratifying by household size and the duration between symptom onset/confirmation and isolation or hospitalization of the index case. We explored age-dependent differences in infectivity and susceptibility across all contact locations and household contacts (stratified by ages 0–19 years and 20+ years). Finally, we examined differences in transmissibility by symptom status of index cases across all exposure locations because of limited studies stratifying by symptom status and exposure location. Stratifications were chosen to maximize the available data across studies.

Because of potential within-study correlation (eg, individuals in the same locations experiencing the same public health interventions and country-specific home, travel, and work practices) and between-study heterogeneity from study and population differences, we employed a beta-binomial model to pool SARs and a Poisson-gamma mixture model to pool R_obs_ across studies (Supplementary Methods) [7–9].

## RESULTS

**Figure 1.**
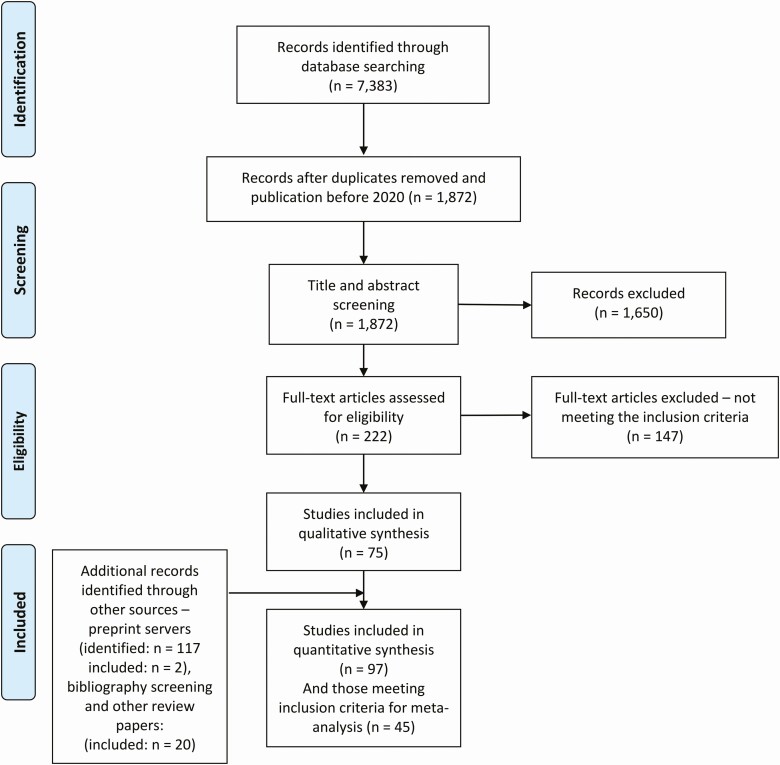
PRISMA flow diagram of study selection.

We identified 1872 published studies, 75 of which were included after full-text screening (Figure 1). A further 22 eligible studies were identified through preprint servers and bibliography screening. Of these 97 studies, summarized in Supplementary Tables 1–7, 67 tested all contacts regardless of symptom status. Among those, 45 reported data from >1 index case and were included in the meta-analysis.

### Household

We identified 29 eligible studies reporting household contacts [10–38], with more than one-half carried out in China (Supplementary Table 1). Household definitions were broadly consistent across studies, requiring contacts to be living in the same residence as the index case. One study additionally included nonresident contacts who spent ≤24 hours in the same residence as the index case [22] (Supplementary Table 1), but its inclusion had no significant impact on the pooled SAR estimate.

**Figure 2.**
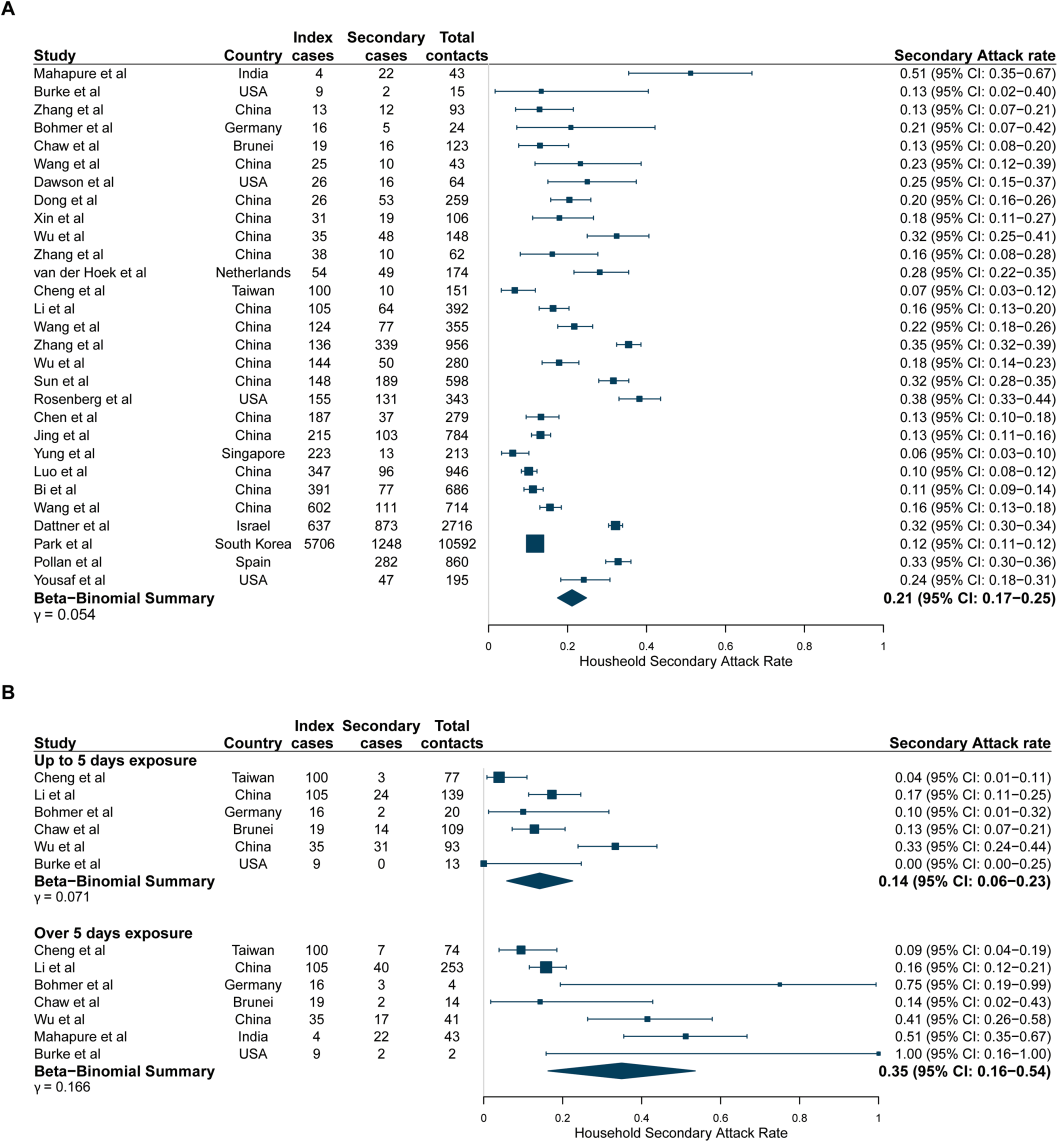
(*A*) Pooled overall household secondary attack rates. Studies are ordered by the number of index cases reported in the study. Studies of large household contact tracing investigations were included regardless of whether the number of index cases was reported in the study. (*B*) Stratified by duration of household exposure to symptomatic index case. Studies are ordered by the number of index cases reported in the study because this information was not given by exposure duration. Study-level point estimates and binomial confidence intervals are shown along with the pooled beta-binomial summary across studies. Exposure duration to the index case before isolation or hospitalization categories (≤5 days and >5 days) were selected to maximize usage of data.

Study estimates of household SARs ranged from 6.1% [25] to 51.2% [38] with a pooled estimate of 21.1% (95% confidence interval [CI]: 17.4–24.8) (Figure 2A). The relationship between the number of secondary cases and the number of index cases is shown in Supplementary Figure 1. R_obs_ varied across studies from 0.05 [25] to 5.5 [38], with a pooled household R_obs_ of 0.96 (95% CI: 0.67–1.32) (Supplementary Table 8). The SAR increased with longer durations of exposure (14.2% [95% CI: 5.8–22.5] with ≤5 days of exposure to an index case vs 34.9% [95% CI: 16.3–53.6] with >5 days of exposure; *P* = .05; Figure 2B). Longer durations of exposure were similarly associated with an increased pooled R_obs_ (0.40 [95% CI: 0.21–0.72] with ≤5 days to 1.91 [95% CI: 0.86–3.55] with >5 days, *P* < .001) (Supplementary Table 9). There was a trend for decreasing transmission with increasing household size, but this was not statistically significant (*P* = .29) (Supplementary Figure 2). Study-level estimates for the small subset of studies reporting household sizes were highly variable.

### Workplaces

**Figure 3.**
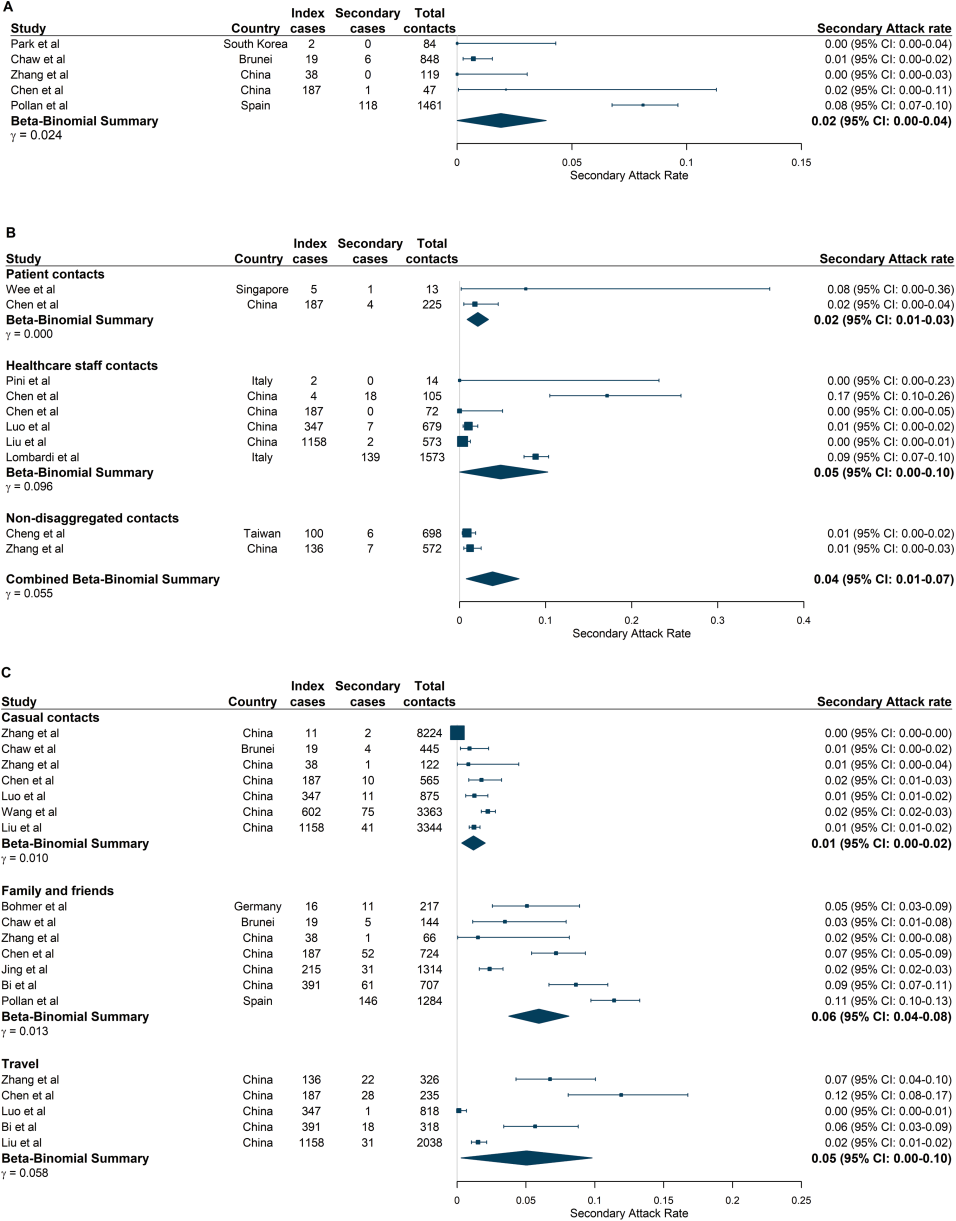
Secondary attack rates stratified by exposure locations. (*A*) Workplace-based contacts. (*B*) Healthcare-based contacts. Beta-binomial summary estimates are presented for patient and healthcare staff contacts of index cases and a combined (nondisaggregated) contacts category. This “combined contacts” summary estimate was pooled across all studies in the healthcare setting and includes 2 studies in which disaggregated contact groups were not reported. (*C*) Social contact environments. Studies are ordered by the number of index cases reported at the study level. Large population-level studies were included irrespective of whether the number of index cases was reported by the study. Abbreviation: CI, confidence interval.

Seven studies reported workplace contacts (Supplementary Table 3) [13, 27, 28, 31, 39–41]. Three of these studies were cluster investigations from a single index case, including a boardroom meeting in Germany (SAR: 91.7% [95% CI: 61.5–99.8], 11/12) [41], supermarket employees in China (SAR: 9.2% [95% CI: 4.7–15.8], 10/120) [28] and call center colleagues in South Korea (SAR: 43.5% [95% CI: 36.8–50.4], 94/216) [40]. Excluding those, the pooled SAR reduced from 12.3% (95% CI: 1.3–22.5) (Supplementary Figure 3) to 1.9% (95% CI: 0.0–3.9) (Figure 3A). Workplace contacts likely represented a variety of sectors across studies, and only 1 study provided a detailed definition [40].

### Healthcare Facilities

Twenty-eight studies reported healthcare-based contacts, 10 of which met our inclusion criteria for meta-analysis (Supplementary Table 4) [30, 33, 39, 42–46], including index case populations of patients [30, 33, 39, 42, 44, 45], healthcare workers [43], or both [21, 46]. Study-level SARs varied from 0.0% to 17.1%, resulting in a pooled estimate among any healthcare contacts of 3.6% (95% CI: 1.0–6.9) (Figure 3B). There was no significant difference in the pooled SAR between patient or healthcare staff contact subgroups (*P* = .64; Figure 3B). The estimated R_obs_ in healthcare settings was 1.18 (95% CI: 0.65–2.04) but ranged between 0.0 and 4.5 (Supplementary Table 10, Supplementary Figure 1).

### Social Settings

Twenty-two studies were identified with social contact settings including travelling, religious events, fitness classes, shopping, entertainment venues, and events with family and friends (Supplementary Table 6). Of these, 13 fulfilled the criteria for meta-analysis [10, 11, 13, 17, 26–28, 30, 31, 37, 39, 45, 47]. We estimate low SARs in low-contact events with casual contacts or strangers, with a pooled SAR of 1.2% (95% CI: 0.3–2.1) (Figure 3C). In contrast, in settings with more familiar and prolonged contact such as events with family and friends, the pooled SAR was 5.9% (95% CI: 3.8–8.1) and R_obs_ was 0.38 (95% CI: 0.18–0.64) (Supplementary Table 11, Supplementary Figure 1). Travel-related contacts had an estimated SAR of 5.0% (95% CI: 0.3–9.8), similar to that in social events with family and friends (Figure 3C, Supplementary Table 6). R_obs_ could not be estimated for any other contact groups because of insufficient data. Several other social settings with high levels of transmission were identified but could not be pooled (Supplementary Results).

### Exposure Location Summary

Table 1 summarizes the pooled estimates of SAR and R_obs_ across different exposure locations. Although the highest SARs were estimated for household contacts and in familial settings, the highest R_obs_ was estimated for healthcare settings. Pooled estimates for schools and care homes were not possible because of scarcity of studies (Supplementary Results).

**Table 1.** Summary Table of the Pooled SAR and R_obs_ for the Exposure Locations Considered in this Study

Setting	Pooled SAR (%)	95% CI	Pooled R_obs_	95% CI
Households	21.1	17.4–24.8	0.96	0.67–1.32
Social gatherings with family and friends	5.9	3.8–8.1	0.38	0.18–0.64
Travel	5.0	0.3–9.8	…	…
Healthcare	3.6	1.0–6.9	1.18	0.65–2.04
Workplace	1.9	0.0–3.9	…	…
Casual close contacts	1.2	0.3–2.1	…	…

Where values are missing, there were not enough data available to estimate a pooled value.

Abbreviations: CI, confidence interval; R_obs_, observed reproduction numbers; SAR, secondary attack rate.

### Age Effects

**Figure 4.**
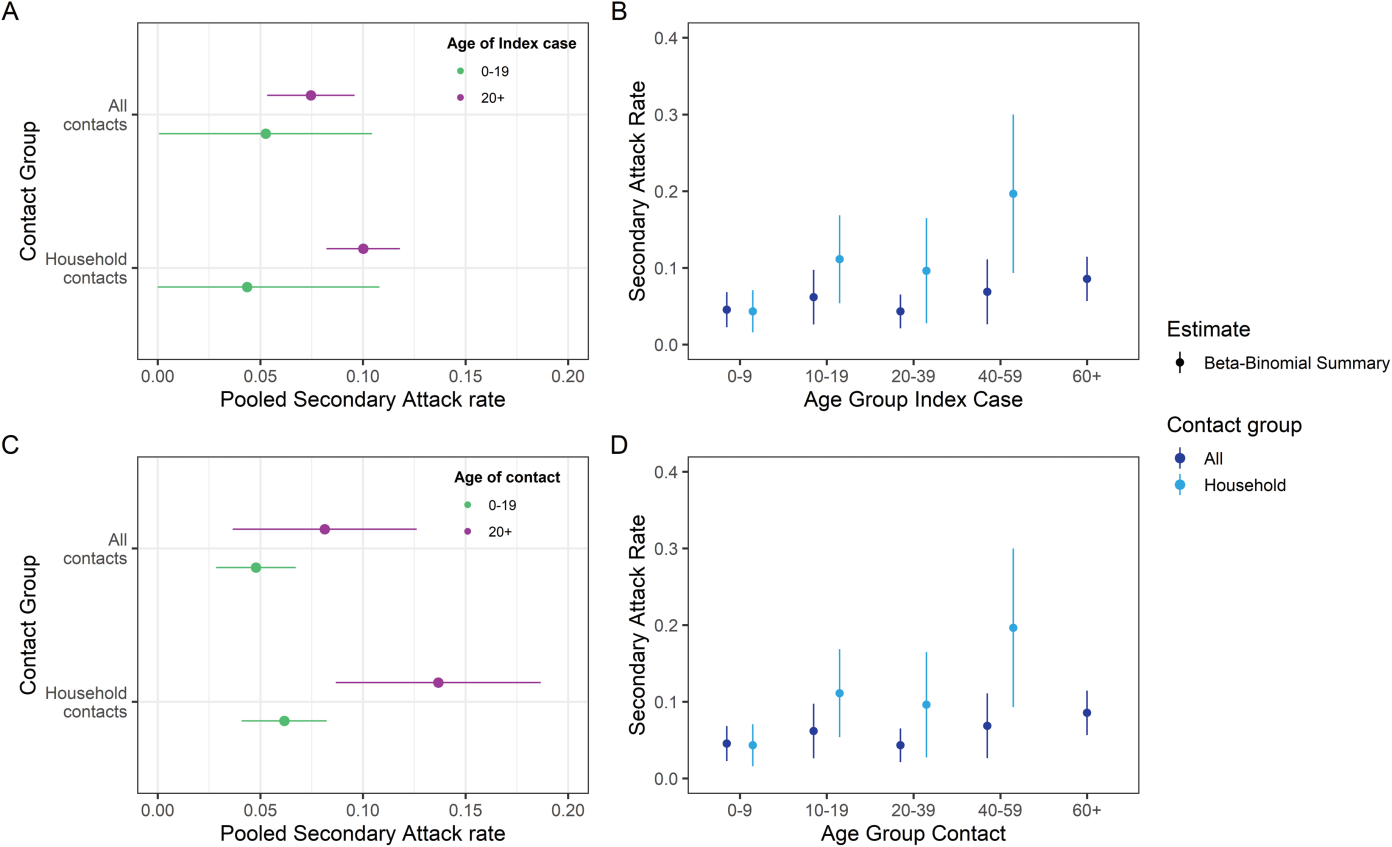
Pooled estimates of secondary attack rates by age of the index case and contacts stratified by contact location. (*A*) Index cases stratified by 0–19 and 20+ year age brackets and exposure location to the index case. (*B*) Index cases stratified by 10-, 20-, and 40-year age brackets. (*C*) Contacts stratified by 0–19 and 20+ year age brackets and (*D*) exposure location contacts stratified by 10-, 20-, and 40-year age brackets. Point estimates were obtained from fitting a beta-binomial model to pooled study data, with 95% confidence intervals shown by horizontal and vertical bars. All contacts combine studies regardless of exposure locations, and household only those studies relating to household transmission. The pooled household secondary attack rates for ages 60+ is not shown because there were insufficient data for this age group.

Ten studies provided age breakdowns of index cases and contacts [10, 12, 16, 22, 25, 31, 33, 37, 45, 48] meeting the inclusion criteria. Pooling across all exposure locations, we found no significant differences in transmissibility or susceptibility by age of index cases or contacts (Figure 4 and Supplementary Table 12, Supplementary Figures 4–5).

However, we observed weak evidence for a difference in transmissibility and a significant difference in susceptibility by age in household studies (index 0–19 years: 4.4% [95% CI: 0.0–10.8] vs index ≥20 years: 10.0% [95% CI: 8.2–11.8], *P* = .07 and contact 0–19 years: 6.16% [95% CI: 4.1–8.2% vs contact ≥20 years: 13.7% [95% CI: 8.7–18.7], *P* = .01) (Figure 4A, Figure 4C). When further disaggregating age groups, the trend for SARs to increase with increasing age of index cases and contacts was maintained (Figure 4B, Figure 4D). We note that, across contact age groups, the SARs increased at a higher rate for household contacts (Figure 4D).

### Symptom Status of Index Case

**Figure 5.**
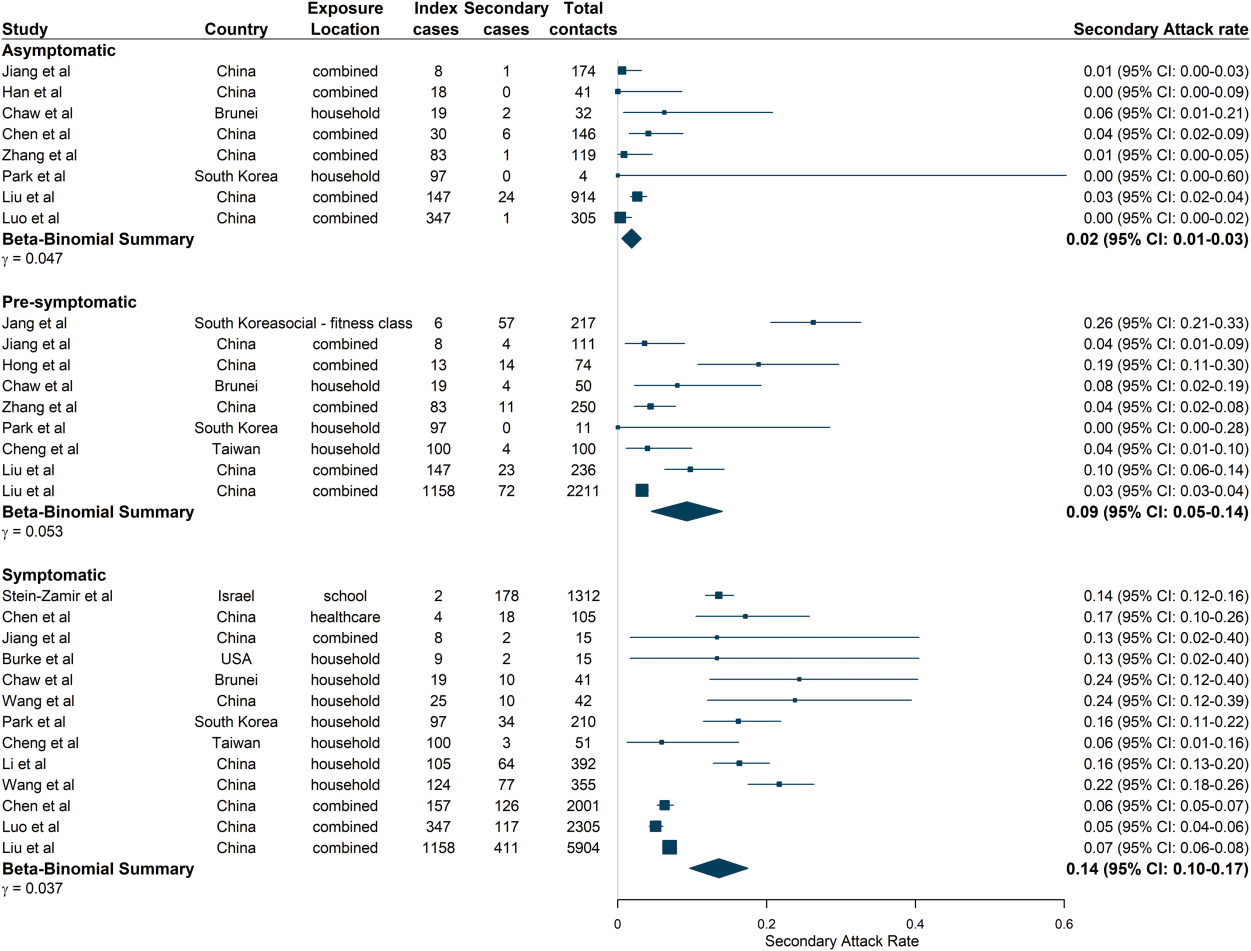
Estimated secondary attack rates from asymptomatic, presymptomatic, and symptomatic index cases. Studies are ordered by the number of index cases reported in the study as shown in the figure. Pooled estimates combine all exposure locations listed. Combined exposure locations relate to contact tracing studies where close contacts were not disaggregated by exposure location. Asymptomatic index cases were defined as those with a positive severe acute respiratory syndrome coronavirus 2 reverse transcriptase-polymerase chain reaction test and no reported clinical symptoms up to discharge or end of follow-up (at least 14 days). Presymptomatic index cases were defined as those not reporting symptoms at the time of testing or during exposure but later developed symptoms. Symptomatic index cases reported coronavirus disease 2019–associated symptoms at the time of sampling and/or during exposure. Abbreviation: CI, confidence interval.

From 18 studies [18, 19, 22, 27, 29–31, 33, 39, 40, 44, 45, 47, 49–53], SAR was lowest for asymptomatic index cases at 1.9% (95% CI: 0.5–3.1) (Figure 5) (asymptomatic definitions and follow-up durations for included studies shown in Supplementary Table 13). SARs were significantly higher for presymptomatic and symptomatic index cases, estimated at 9.3% (95% CI: 4.5–14.0, *P* = .01) and 13.6% (95% CI: 9.7–17.5, *P* < .001), respectively (Figure 5), with similar patterns observed for pooled R_obs_ estimates. Asymptomatic index cases resulted in the lowest R_obs_ of 0.17 (95% CI: 0.04–0.45), followed by presymptomatic index cases (R_obs_ = 0.78 [95% CI: 0.36–1.44], *P* = .004) and symptomatic index cases (R_obs_ = 1.01 [95% CI: 0.57–1.61], *P* < .001) (Supplementary Table 14, Supplementary Figure 1).

## DISCUSSION

This systematic review provides an indication of the types and places of contacts and index case/contact characteristics that facilitate SARS-CoV-2 transmission. We found evidence to suggest that more familiar prolonged contact increases the potential for transmission, as does the presence or potential for symptoms with moderate age-dependent effects observed at the household level. However, there were limited data to allow exploration of transmission patterns in workplaces, schools, and care homes.

Sustained daily contact within households may explain the high SAR (21.1%), which is comparable to the pooled estimate of an earlier systematic review (18.1%) [5]. Our estimate hides substantial heterogeneity between studies, with reported SARs ranging between 0% and 51%. This is similar to influenza household transmission, where SARs ranged from 1% to 38% [54, 55], but higher than those for SARS-1 (6%–8%) [56,57]. We found evidence of reduced SARs and R_obs_ when index cases were isolated within 5 days of symptom onset, suggesting that household transmission characteristics are influenced by contact tracing and isolation policies. Although SARs could not be estimated in other residential settings, it is not unexpected that these locations report high study-specific attack rates. Elderly residents living in care homes are particularly vulnerable populations, with high risk of severe outcomes and coronavirus disease 2019 (COVID-19)-related mortality [2, 3], and. Understanding dynamics of transmission in these environments is vital to prevent further outbreaks. With many countries continuing to recommend “stay-at-home” measures, with cases isolating inside households, it is likely that this location will continue to be important in sustaining transmission.

We estimated a relatively low SAR (3.6%) in healthcare settings, which is substantially lower than that observed during the early stages of the SARS-1 epidemic [58–60]. This is potentially driven by the large number of identified and tested contacts per index case and stricter measures such as mask mandates and austere visitor policies. Although the probability of onward infection per individual contact is low in healthcare settings, the high number of contacts can lead to more opportunities for infection, which may explain the higher R_obs_ (1.18) in this setting. It is plausible that sufficient infection control and personal protective equipment (PPE) limited transmission potential in these settings but could not be explored because of insufficient data. Notably, all studies took place in high- and middle-income countries where PPE is likely more widely available which, in addition to the diversity of these healthcare systems, make generalizations about SARS-CoV-2 transmission rates in healthcare facilities difficult.

Despite a relatively low transmission potential in index cases who do not develop symptoms (SAR 1.9%), our findings suggest that SAR from exposure to presymptomatic index cases is similar to that of symptomatic cases (9.3% vs 13.6%, *P* = .08). The difficulty in identifying presymptomatic cases, who have been estimated to account for 47% of onward transmission [61], is particularly challenging for control policies. Our results highlight the continued need for physical distancing policies and widescale testing and contact tracing to identify and isolate those cases not yet showing symptoms. With asymptomatic transmission rates estimated to be significantly lower, this could have positive implications for vaccines that protect against symptomatic disease, potentially reducing the risk of transmission also.

Although studies reporting on the age breakdown of index cases and contacts were limited, we found evidence for age dependence in transmissibility and susceptibility in the household context, which was more limited when pooling across all settings. There is significant uncertainty around these estimates because of the sparsity of data. Therefore, understanding potential age-dependent effects in transmissibility and susceptibility remains challenging. Observed age differences could relate to behavioral, contextual, or biological factors that are not yet fully understood [62]. There is growing evidence to suggest that children tend to experience mild or asymptomatic infections [63–67], which may explain the potential differences observed in SAR from patients younger than 20 years of age. Further to this, symptomatic testing policies may fail to detect childhood infections or identify children as index cases, which could bias estimates.

In contrast to influenza, in which schools are clearly important contributors to transmission [68, 69], school-based studies on SARS-CoV-2 are limited because of reactive school closures. In addition to schools closing, many countries have and continue to encourage work-from-home practices. Furthermore, workplace settings vary greatly not just by country, but also between sectors, with some more able to facilitate COVID-19 safety measures, highlighting the difficulty in ascertaining a universal workplace SAR. Currently, there is insufficient information to explore SARs in different types of schools, workplaces, or sectors.

We estimate relatively low R_obs_ across all settings, suggesting that the use of contact tracing and isolation activities in these studies is an effective measure at reducing onward transmission. However, low numbers of studies were pooled and therefore there was considerable uncertainty in these estimates. Additionally, large cluster outbreaks can occur, as evidenced in several studies [28, 31, 38, 40, 41, 47, 70–73] and often, these events have the potential to overwhelm surveillance systems. The aggregate nature of the data used for our R_obs_ estimates likely hides substantial individual-level heterogeneity in transmission potential, hindering estimation of the overdispersion factor relating to SARS-CoV-2 transmission. Characterizing this potential for “super-spreading events” will be critical for further informing where to direct contact tracing efforts. Early evidence suggests that transmission is overdispersed with around 19% of cases resulting in 80% of transmission in Hong Kong [74] and modeling studies based on reported global cluster sizes that suggested that as few as 10% of cases could account for 80% of all SARS-CoV-2 transmission [75]. The large cluster outbreaks identified in this review tend to be reported in indoor social or workplace settings, potentially highlighting these locations as facilitating super-spreading events [76].

There are several limitations to this study. As in any active outbreak response, data collected from multiple teams under different country guidelines are subject to variations in definitions, follow-up time, and testing protocols, limiting interpretation of pooled data. Furthermore, individual studies suffer from reporting and recall bias in identifying all contacts of a confirmed case. Across settings, index cases were commonly enrolled after presenting for medical attention and therefore can be biased toward infections that result in symptomatic or more serious illness, potentially distorting transmission pathways [54]. Without phylogenetic sequencing, it is difficult to confidently resolve these pathways which could bias SARs estimates; for example, if the index case was asymptomatic and is instead identified as a secondary case of the symptomatic case that first presented for testing or if transmission occurs outside the household but is attributed to a household index case. Globally, there is still a lack of detailed data from contact tracing studies to fully explore differing transmission potential by more precise exposure locations or individual characteristics. In this meta-analysis, the majority of studies came from China, where strict control policies were implemented, possibly limiting the generalizability of estimates. In addition, these studies were performed before the identification of new virus variants with potentially enhanced transmission potential [77]. It will be important to contrast our estimates with studies distinguishing between variants when available. Finally, evidence is continually emerging from newspaper articles, government reports, and press conferences that report on large outbreaks in care homes, schools, workplaces, and hospitals. Such sources cannot be systematically searched or reliably cited, and this information is not translated into published contact tracing studies because of limited public health resources and the prioritization of the ongoing emergency response.

In conclusion, early data suggest that SARS-CoV-2 transmission is highest in locations in which sustained and prolonged contacts are made, including households and other residential locations. With many countries continuing to issue stay-at-home measures, ensuring sufficient and early isolation of cases from other household members will be crucial to prevent spread. The identified similar transmission potential from presymptomatic and symptomatic cases is also challenging for control policies and highlights the importance of enhanced contact testing, that is not reliant on symptom status, rapid isolation, and physical distancing of both symptomatic and asymptomatic cases, as well as the continued use of appropriate PPE. Further research on transmission in different social settings, as well as in schools and workplaces, in which there are limited data to date, is required to continue to inform transmission reduction strategies.

## Supplementary Data

Supplementary materials are available at *Clinical Infectious Diseases* online. Consisting of data provided by the authors to benefit the reader, the posted materials are not copyedited and are the sole responsibility of the authors, so questions or comments should be addressed to the corresponding author.

ciab100_suppl_Supplementary_Materials

## Notes

***Acknowledgments.*** The authors thank the following authors for providing information on primary studies included in this review and extracting additional relevant data: Drs. Rachel Burke, Hao-Yuan Cheng, Tasuku Hara, and Hsien-Ho Lin, and all of their colleagues involved in the original publications. The authors acknowledge all members of the Imperial College COVID-19 Response Team.

***Disclaimer.*** The funders had no role in study design, data collection and analysis, decision to publish, or preparation of the manuscript. The views expressed are those of the authors and not necessarily those of the United Kingdom (UK) Department of Health and Social Care, the National Health Service, the National Institute for Health Research (NIHR), Public Health England (PHE), UK MRC, UKRI, or European Union.

***Financial support*.** This work was supported by the National Institute for Health Research Health Protection Research Unit (NIHR HPRU) in Modelling and Health Economics, a partnership between Public Health England, Imperial College London and the London School of Hygiene and Tropical Medicine (grant number: NIHR200908). We acknowledge funding from the Medical Research Council (MRC) Centre for Global Infectious Disease Analysis (grant number: MR/R015600/1), jointly funded by the UK Medical Research Council and the UK Foreign, Commonwealth & Development Office (FCDO), under the MRC/FCDO Concordat agreement and is also part of the EDCTP2 program supported by the European Union. In addition, H. A. T. and A. M. are supported by a UK Medical Research Council Studentship (grant number: G98669).

***Potential conflicts of interest.*** A. C. G. reports grants from Medical Research Council during the conduct of the study; grants from Bill and Melinda Gates Foundation, Wellcome Trust, National Institutes of Health, PATH, Medicines for Malaria Venture, and Integrated Vector Control Consortium and personal fees from Malaria programming from The Global Fund, outside the submitted work. N. M. F. reports serving as principal investigator of a grant for the MRC Centre for Global Infection Disease Analysis (UK Medical Research Council), a grant funding the NIHR Health Protection Research Unit for Mathematical Modelling and Health Economics (UK National Institute of Health Research), and a grant funding the Jameel Institute of Disease and Emergency Analytics at Imperial College (Community Jameel), during the conduct of the study. N. M. F. also reports serving as principal investigator of a National Institutes of Health U01 grant under the NIGMS MIDAS program (NIH NIGMS), as the co-investigator on a grant modeling antivirals against dengue (Janssen Pharmaceuticals), as the principal investigator of 2 grants from the Bill and Melinda Gates Foundation, including one funding the Vaccine Impact Modelling Consortium (Bill and Melinda Gates Foundation), and a grant from Gavi, funding the Vaccine Impact Modelling Consortium (Gavi, the Vaccine Alliance), outside the submitted work. All other authors declare no conflicts of interest. All authors have submitted the ICMJE Form for Disclosure of Potential Conflicts of Interest. Conflicts that the editors consider relevant to the content of the manuscript have been disclosed.
