# Supplementary material for: Severe Acute Respiratory Syndrome Coronavirus 2 (SARS-CoV-2) Setting-specific Transmission Rates: A Systematic Review and Meta-analysis
Source: Clin Infect Dis. 2021 Feb 9;73(3):e754–64. doi: 10.1093/cid/ciab100 (PMC7929012; doi:10.1093/cid/ciab100)
Supplement: ciab100_suppl_Supplementary_Materials [file ciab100_suppl_supplementary_materials.docx]

**Supplementary Material**

**SARS-CoV-2 setting-specific transmission rates: a systematic review and meta-analysis**

Hayley A Thompson^1*^, Andria Mousa^1^, Amy Dighe^1^, Han Fu^1^, Alberto Arnedo-Pena^2,3^, Peter Barrett^4,5^, Juan Bellido-Blasco^2,3,6^, Qifang Bi^7^, Antonio Caputi^8^, Liling Chaw^9^, Luigi De Maria^8^, Matthias Hoffmann^10^, Kiran Mahapure^11^, Kangqi Ng^12^, Jagadesan Raghuram^12^, Gurpreet Singh^13^, Biju Soman^13^, Vicente Soriano^14^, Francesca Valent^15^, Luigi Vimercati^8^, Ian Wee^16^, Justin Wong^17,9^, Azra C Ghani^1^, Neil M Ferguson^1*^

1. MRC Centre for Global Infectious Disease Analysis & WHO Collaborating Centre for Infectious Disease Modelling, Abdul Latif Jameel Institute for Disease and Emergency Analytics, Imperial College London, London, UK
2. Sección de Epidemiología. Centro de Salud Pública de Castellón, Valencia, Spain
3. Centro de Investigación Biomédica en Red de Epidemiología y Salud Pública (CIBERESP), Valencia, Spain
4. School of Public Health, University College Cork, Cork, Ireland
5. Irish Centre for Maternal and Child Health Research (INFANT), University College Cork, Cork, Ireland
6. Facultad de Ciencias de la Salud, Universitat Jaime I (UJI), Castelló, Spain
7. Department of Epidemiology, Johns Hopkins Bloomberg School of Public Health, Baltimore, MD, USA
8. Interdisciplinary Department of Medicine, University of Bari, Unit of Occupational Medicine, University Hospital of Bari, Bari, Italy
9. PAPRSB Institute of Health Sciences, Universiti Brunei Darussalam, Jalan Tungku Link, Brunei
10. Division of General Internal Medicine, Infectious Diseases and Hospital Epidemiology, Cantonal Hospital Olten, Switzerland
11. Department of Plastic Surgery, Dr Prabhakar Kore Hospital and MRC, Belgaum, Karnataka, India
12. Changi General Hospital, Singapore
13. Sree Chitra Tirunal Institute for Medical Sciences and Technology, Thiruvananthapuram, Kerala, India
14. UNIR Health Sciences School & Medical Center, Madrid, Spain
15. SOC Istituto di Igiene ed Epidemiologia Clinica, Azienda Sanitaria Universitaria Friuli Centrale, Udine, Italy.
16. Department of Infectious Diseases, Singapore General Hospital, Singapore, Singapore
17. Disease Control Division, Ministry of Health, Brunei

## Supplementary Methods

We did not exclude articles based on quality scoring given the emergency response context within which the studies were conducted; articles that met the inclusion criteria for meta-analysis all had high quality scores and therefore no sensitivity analysis was conducted.

For SAR estimates, we assume that the distribution of secondary cases $x_{i}$ from $n_{i}$ close contacts for study $i$ is binomially distributed according to: $x_{i} \sim Bin\left( n_{i},p_{i} \right)$, conditional on probability $p_{i}$ (the SAR). When $p_{i}$ varies across studies, a single binomial distribution cannot adequately describe this additional variation. To account for this, we assume that $p_{i}$ is drawn from a beta distribution which describes how attack rates from different studies relate to each other: $p_{i} \sim Beta\left( \alpha,\beta\right).$Reparametrizing the beta distribution in terms of its mean$: \mu=\alpha/\left( \alpha+ \beta\right),$ and variance:$\theta=1/\left( \alpha+ \beta\right)$ enables fitting this model to the pooled study data using maximum likelihood methods according to the log-likelihood equation:

$$L\left( \mu,\theta\right|x,n)= \sum_{i=1}^{k} \left[ \sum_{r=0}^{x_{i}-1} \log\left( \mu+r\theta\right)+ \sum_{r=0}^{n_{i}-x_{i}-1} \log\left( 1-\mu+r\theta\right)- \sum_{r=0}^{n_{i}} log(1+r\theta) \right]$$

Where $i$and $r$ are running indexes, and *k* is the total number of studies included [1]. We report the mean of the resulting beta-binomial distribution and its corresponding 95% Confidence Interval as the pooled SAR across studies. The parameter $\gamma$ of the beta-binomial distribution is defined as $\theta/(1+\theta)$, $0 \leq\gamma\leq1$, and is a measure of the variability in the study data. With 0 indicating no overdispersion and 1 highly over dispersed data. An advantage of the beta-binomial is that no transformations of the data are required and therefore no continuity corrections are needed to cope with zero numerators.

A similar approach was taken when estimating R_obs_ across studies employing a Poisson-Gamma model. Here we assume that the observed number of secondary cases $x_{i}$ from study $i$ is Poisson distributed according to: $x_{i} \sim Poisson\left( n_{i}\lambda_{i} \right)$ where $n_{i}$ is the number of index cases per study and $\lambda_{i}$ is the study-specific R_obs_. We account for variation in R_obs_ across studies by assuming that $\lambda_{i}$ is drawn from a gamma distribution: $\lambda_{i} \sim Gamma\left( \alpha,\beta\right)$ with mean $\frac{\alpha}{\beta}$ equating to the pooled estimate of R_obs_ across studies. This model is fitted to the pooled study data using maximum likelihood methods according to the log-likelihood equation:

$$L\left( \alpha,\left. \beta\right|x_{i},n_{i} \right)=\sum_{i=1}^{k} \log\frac{\Gamma\left( x_{i}+\alpha\right)}{\Gamma\left( a \right)}+\alpha\log\left( \beta\right)-\left( x_{i}+\alpha\right)\log\left( n_{i}+\beta\right)$$

Significance tests were conducted to test for differences between sub-group means for both transmission metrics using likelihood ratio tests [1–3]. All analysis was conducted using R version 3.6.3.

## Supplementary Results

## Schools

Studies reporting on school-based contacts of SARS-CoV-2 positive individuals were limited (Table 2). We identified seven studies [4–10] of which only 2 tested all contacts regardless of symptom status, one from a military school in Switzerland (recruit index case)[4] which reported a SAR of 1.8% (95% CI: 0.1%-9.7%, 1/55) and one from a high school in Israel (two student index cases)[10] reporting a SAR of 13.2% (95% CI:11.3%-16.9%, 153/1161) in student contacts and 16.6% (95% CI: 11.0%-23.5%, 25/151) in staff contacts. In both studies schools were open (pre-lockdown and post-lockdown respectively) and index cases were symptomatic during their attendance. There is limited statistical power from these two studies to estimate a representative school-based SAR.

An additional study from Australia tested only symptomatic individuals and a subset (around one third) of identified student and staff contacts across nursery, primary and high schools [9]. Eighteen secondary cases were identified from 27 index cases (12 adults and 15 children). However, during the study period, attendance at schools reduced from 90% to 5% with cases in children attending school peaking in late March when attendance had rapidly reduced which limits exploration of transmission potential in these settings [9]. Two further studies from Ireland [8] and France [5] tested only symptomatic school contacts but found no evidence on onward transmission in these instances to either students or staff. The final two studies pertain to serosurveys conducted in French primary and secondary schools with known attendance at these schools of students and staff infected with SARS-CoV-2. While the data from these studies cannot be used to estimate SARs, they highlight the potential for a large overall attack rate in school aged children: high school students 92/242 with antibodies (attack rate 38.0% [95% CI: 31.8%-44.4%]) [6] and 43/530 from a primary school student population (attack rate 8.11% [95% CI: 5.9%-10.7%]) [7]. However, for those studies, it is not clear if transmission occurred within the schools while they were open or outside the school setting. The authors noted that prior to school closures, three infected pupils attended different primary schools with no secondary cases in the following 14 days, however the duration of attendance at school and the symptom status of these children are not reported [7].

## Group Living Populations

In this section we describe settings where individuals tend to live in close proximity, often with limited opportunity to social distance. There was limited data available on these settings in the published and pre-print literature, with only six studies identified. Three of these studies were conducted in care homes, located in Washington USA (Table 5), following the identification of one [11] or two [12,13] index cases living in the care home. However only two studies tested all resident contacts regardless of symptom status. These reported attack rates of 30.3% (95% CI: 20.2%-41.9%) [11] and 3.8% (95% CI: 0.8%-10.6%) [12]. The third study only tested 118 of around 170 residents but reported an attack rate of 59.4% (95% CI 51.6%-66.9%, 101/170)[13]. There were important differences between the studies included in this meta-analysis that may explain the variability observed in different facility-level attack rates. In the study by Roxby *et al*, the residential facility implemented high levels of social distancing among residents and low contact with healthcare providers following the identification of an index case [12]. Furthermore, both index cases were rapidly isolated. This likely resulted in lower overall transmission in this facility compared with that presented in Kimball *et al* and McMichael *et al,* both from skilled nursing facilities where residents were more likely to be in shared rooms with prolonged close contact with healthcare providers [11,13].

Another vulnerable population living in group accommodation are the homeless. Only one published study was conducted in a homeless shelter population in Boston, USA following the identification of increasing numbers of cases associated with the facility [14]. This study reports a high attack rate of 36.0% (95% CI: 31.4%-40.8%) with 147/408 residents testing positive, 87.8% of which were asymptomatic [14]. We also identified a study conducted in a military training base, where recruits lived in close quarter barracks. This study did not test all close contacts but only those who were symptomatic [15]. Among 85 tested close contacts from a single index case (from a population of 4,071), 3 additional cases were identified [15]. Finally one study from Louisiana USA reported on SARS-CoV-2 transmission in prison populations, where 489 confirmed cases were detected among 46 different facilities in the State [16]. However, not all facilities implemented or reported on testing of all contacts regardless of symptom status and therefore power to understand transmission rates in these locations is limited. We did not identify any studies looking at other population groups that live in crowded settings such as in refugee camps or migrant communities.

## Social Settings

The following details studies identified in the systematic review that described contact investigations from social settings but could not be pooled in the meta-analysis. Two studies reported on transmission from large group faith-based events. The first was a religious pilgrimage over the course of 8 days which resulted in an attack rate of 90.6% (95% CI: 79.3%-96.9%) following the identification of two primary cases with 48/53 of the group testing positive [17]. The second was related to a local religious gathering in Brunei which ran throughout the night with participants staying overnight and reported an attack rate of 14.8% (95% CI: 6.6%-27.1%)[18]. Lastly, transmission events related to fitness dance classes in South Korea where 6 pre-symptomatic instructors taught several classes resulting in 57 secondary cases and a SAR of 26.3% (95% CI: 20.5%-32.7%) and a high study R_obs_ of 9.5 (95% CI: 4.8-17.1) [19]. Three clusters were also reported from social event settings resulting from single index cases (and thus likely to be subject to publication bias) – a ski chalet cluster which was one of the first reports of transmission in Europe where 11/15 friends and families sharing an apartment were infected by a single index case (SAR 73.3% [95% CI: 44.9%-92.2%]) [5], a wedding in Jordan where 76/350 guests tested positive following interaction with the symptomatic father of the bride (SAR 21.7% [95% CI: 17.5%-26.5%]) [20] and a social meeting between friends including a dinner and karaoke with an asymptomatic index case which resulted in 7/17 friends testing positive (SAR 41.1% [95% CI: 18.4%-67.1%]) [21]. These studies all highlight the potential for large clusters to be associated with transmission from a single index case suggesting social settings could facilitate large super spreading events.

## Supplementary Tables

## Households

**Table 1. Summary of studies reporting on household contacts of index cases.** Where index cases were not broken down by exposure location the study level number of index cases is provided.

| **Main text reference number** | **Author** | **Region** | **Country** | **Study design** | **Contact definition** | **Testing strategy** | **Testing method** | **Index cases** | **Contacts traced** | **Contacts tested** | **Secondary cases** |
| --- | --- | --- | --- | --- | --- | --- | --- | --- | --- | --- | --- |
|  | Arnedo-Pena et al [22] | Castellon | Spain | Retrospective cohort study | close contacts living in the same household with the index case | symptomatic | RT-PCR | 347 | 745 | 745 | 83 |
|  | Bai et al [23] | Gansu | China | Familial cluster investigation | Lived in the same household with the index case | all | RT-PCR | 1 | 6 | 6 | 5 |
| 10 | Bi et al [24] | Shenzhen | China | Surveillance and contact tracing | Lived in the same apartment with an index case 2 days before symptom onset | all | RT-PCR | 391 | 686 | 686 | 77 |
| 11 | Bohmer et al [25] | Bavaria | Germany | Outbreak investigation and contact tracing | Sharing living space with the index case | all | RT-PCR | 16 | 24 | 24 | 5 |
| 22 | Burke et al [26] | Not specified | USA | Surveillance and contact tracing | Family members/friends of a travel-associated case patient who spent at least one night in the same residence during the presumed infectious period | all | RT-PCR | 9 | 15 | 15 | 2 |
| 31 | Chaw et al [18] | Not specified | Brunei | Cluster investigation and contact tracing | Lived in the same household with the index case | all | RT-PCR | 19 | 123 | 123 | 16 |
|  | Chen et al [27] | Xiangyang | China | Familial cluster investigation | Family members who lived in the same household as the index case | all | RT-PCR | 1 | 3 | 3 | 2 |
| 39 | Chen et al [28] | Ningbo | China | Surveillance and contact tracing | Lived in the same household with the index case | all | RT-PCR | 187 | 279 | 279 | 37 |
| 33 | Cheng et al [29] | Not specified | Taiwan | Surveillance and contact tracing | Lived in the same household with the index case | all | RT-PCR | 100 | 151 | 151 | 10 |
| 34 | Dattner et al [30] | Bnei Brak | Israel | Surveillance and contact tracing | Lived in the same household with the index case | all | RT-PCR | 637 | 2,716 | 2,716 | 873 |
| 35 | Dawson et al [31] | Wisconsin | USA | Convenience sample of households with confirmed index case <10 days from diagnosis | Lived in the same household with the index case | all | RT-PCR | 26 | 64 | 64 | 16 |
| 36 | Dong et al [32] | Tianjin | China | Surveillance and contact tracing | Lived in the same household with the index case | all | RT-PCR | 26 | 259 | 259 | 53 |
|  | Draper et al [33] | Northern Territory | Australia | Surveillance and contact tracing | Lived in the same household with the index case | symptomatic | RT-PCR | 28 | 52 | not specified | 2 |
|  | Guallar et al [34] | Madrid | Spain | Cluster investigation | Lived in the same household with the index case | all | serology (all) and RT-PCR (subset) | 1 | 36 | 36 | 32 |
|  | Hu et al [35] | Nanjing | China | Surveillance and contact tracing | Lived in the same household with the index case | all | RT-PCR | 1 | 3 | 3 | 3 |
|  | Hua et al [36] | Zhejiang | China | Retrospective cohort study | Lived in the same household with the index case | subset | RT-PCR | 314 | 1049 | 835 | 151 |
|  | Jiang et al [37] | Shandong Province | China | Epidemiological cluster investigation | Family members who lived in the same household as the index case | all | RT-PCR | 1/2 | 5 | 5 | 2 |
| 37 | Jing et al [38] | Guangzhou | China | Retrospective contact investigation | Unprotected close contact within 1m of index case | all | RT-PCR | 215 | 784 | 784 | 103 |
|  | Laxminaraya et al [39] | Tamil Nadu and Andhra Pradesh | India | Surveillance and contact tracing | Lived in the same household with the index case | subset | RT-PCR | 997 | 4,066 | not specified | 380 |
| 29 | Li et al [40] | Zaoyang and Chibi | China | Retrospective contacts investigation | Household members living at least 24 hrs in the same residence as the index case | all | RT-PCR | 105 | 392 | 392 | 64 |
|  | Li et al [41] | Jiangsu | China | Familial cluster investigation | Lived in the same household with the index case | all | RT-PCR | 1 | 5 | 5 | 4 |
| 32 | Li et al [42] | Wuhan | China | Surveillance and contact tracing | Lived in the same household with the index case | all | RT-PCR/ Serology/Clinical diagnosis | 51 | 120 | 120 | 59 |
| 30 | Luo et al [43] | Guangzhou | China | Surveillance and contact tracing | Lived in the same household with the index case | all | RT-PCR | 347 | 946 | 946 | 96 |
| 38 | Mahapure et al [44] | Maharashtra | India | Surveillance and contact tracing | Family members sharing a large crowded household | all | RT-PCR | 4 | 43 | 22 | 22 |
| 12 | Park et al [45] | Nationwide | South Korea | Outbreak investigation | Lived in the same household with the index case | all | RT-PCR | 5706 | 10,592 | 10,592 | 1,248 |
| 13 | Pollan et al [46] | Nationwide | Spain | Serosurvey | Lived in the same household with the index case | all | Serology | not stated* | 860 | 860 | 282 |
|  | Pung et al [47] | Not specified | Singapore | Cluster investigation and contact tracing | Lived in the same household with the index case | all | RT-PCR | 1 | 4 | 4 | 3 |
| 14 | Rosenberg et al [48] | New York State | USA | Surveillance and contact tracing | Lived in the same household with the index case | all | RT-PCR | 155 | 343 | 343 | 131 |
| 15 | Sun et al [49] | Zhenjiang | China | Surveillance and contact tracing | Lived in the same household with the index case | all | RT-PCR | 148 | 598 | 598 | 189 |
| 16 | van der Hoek et al [50] | Utrecht | Netherlands | Surveillance and contact tracing | Lived at the same residential address | all | RT-PCR | 54 | 174 | 174 | 49 |
| 19 | Wang et al [51] | Wuhan | China | Outbreak investigation and contact tracing | Family members of healthcare workers (index cases) | all | RT-PCR | 25 | 43 | 43 | 10 |
| 18 | Wang et al [52] | Beijing | China | Retrospective cohort study | Family members who lived with primary cases in a house for 4 days before and for more than 24 hours after the primary cases developed illness related to COVID-19 | all | RT-PCR | 124 | 355 | 355 | 77 |
|  | Wang et al [53] | Wuhan | China | Retrospective contact investigation | Lived in the same household with the index case | symptomatic | RT-PCR | 78 | 155 | 104 | 47 |
| 17 | Wang et al [54] | Beijing | China | Surveillance and contact tracing | Lived in the same household with the index case | all | RT-PCR | 602 | 714 | 714 | 111 |
| 20 | Wu et al [55] | Zhuhai | China | Contact investigation/ prospective observational study | Household members living at least 24 hrs in the same residence as the index case after symptom onset | all | RT-PCR | 35 | 148 | 148 | 48 |
| 21 | Wu et al [56] | Hangzhou | China | Retrospective cohort study | Lived in the same household with the index case | all | RT-PCR | 144 | 280 | 280 | 50 |
| 23 | Xin et al [57] | Qingdao | China | Surveillance and contact tracing | Lived in the same household with the index case | all | RT-PCR | 31 | 106 | 106 | 19 |
| 24 | Yousaf et al [58] | Wisconsin, Utah | USA | Surveillance and contact tracing | Lived in the same household with the index case | all | RT-PCR | not stated* | 195 | 195 | 47 |
|  | Yu et al [59] | Xinzhou | China | Surveillance and contact tracing | Lived in the same household or shared a meal with the index case | unclear | unclear | 560 | unclear | unclear | 142 |
| 25 | Yung et al [60] | Not specified | Singapore | Surveillance and contact tracing | Lived in the same household with the index case | all | RT-PCR | 223 | 213 | 213 | 13 |
| 28 | Zhang et al [61] | Liocheng | China | Outbreak investigation and contact tracing | At home family members of index case | all | RT-PCR | 12 | 93 | 93 | 12 |
| 27 | Zhang et al [62] | Guangzhou | China | Surveillance and contact tracing | Lived in the same household with the index case without using proper protection (surgical mask) during 2 days before the index case was tested. | all | RT-PCR | 38 | 62 | 62 | 10 |
| 26 | Zhang et al [63] | Hunan | China | Surveillance and contact tracing | Lived in the same household with the index case | all | RT-PCR | 136 | 956 | 956 | 339 |

* Inferred >1 due to contact sample size

## Schools

**Table 2 Summary of studies reporting on contact investigations in school settings.**

| **Main text reference number** | **Author** | **Region** | **Country** | **Study design** | **Contact definition** | **Testing strategy** | **Testing method** | **Index cases** | **Contacts traced** | **Contacts tested** | **Secondary cases** |
| --- | --- | --- | --- | --- | --- | --- | --- | --- | --- | --- | --- |
|  | Baettig et al [4] | Ticino | Switzerland | Retrospective case series | Contact for >15mins within 2 days of symptom onset of the index patient | all | RT-PCR and serology | 1 | 51 | 51 | 1 |
|  | Danis et al [5] | Not specified | France | Outbreak investigation and contact tracing | All children and teachers who were in the same class as the symptomatic paediatric case | symptomatic | RT-PCR | 1 | 112 | 55 | 0 |
|  | Fontanet at al* [7] | Crepy-en-Valois | France | Retrospective serosurvey | Students of primary schools in Crépy-en-Valois | all | serology | NA | NA | 510 | 43 |
|  |  |  |  |  | Teachers of primary schools in Crépy-en-Valois | all | serology | NA | NA | 42 | 3 |
|  |  |  |  |  | Staff of primary schools in Crépy-en-Valois | all | serology | NA | NA | 28 | 1 |
|  | Fontanet at al* [6] | Oise | France | Retrospective serosurvey | Students of a high schools in Oise | all | serology | NA | NA | 240 | 43 |
|  |  |  |  |  | Teachers of a high schools in Oise | all | serology | NA | NA | 53 | 3 |
|  |  |  |  |  | Staff of a high schools in Oise | all | serology | NA | NA | 28 | 1 |
|  | Heavey et al [8] | Not specified | Ireland | Surveillance and contact tracing | Child and adult contacts who shared activities with 6 index cases in a school setting | symptomatic | RT-PCR | 2 | 1,025 | Not stated | 0 |
|  | Macartney et al [9] | New South Wales | Australia | Surveillance and contact tracing | Secondary school students having face-to-face contact for at least 15 mins or in the same room for 2 hours with a case while infectious | subset | RT-PCR and serology | 12 | 600 | 196 | 2 |
|  |  |  |  |  | Secondary school staff having face-to-face contact for at least 15 mins or in the same room for 2 hours with a case while infectious | subset | RT-PCR and serology | 12 | 96 | 36 | 1 |
|  |  |  |  |  | Primary school and early learning centre students having face-to-face contact for at least 15 mins or in the same room for 2 hours with a case while infectious | subset | RT-PCR and serology | 15 | 585 | 182 | 8 |
|  |  |  |  |  | Primary school and early learning centre students having face-to-face contact for at least 15 mins or in the same room for 2 hours with a case while infectious | subset | RT-PCR and serology | 15 | 167 | 63 | 7 |
| 53 | Stein-Zamir et al [10] | Jerusalem | Israel | Outbreak investigation and contact tracing | Students at a high school in Jerusalem | all | RT-PCR | 2 | 1,164 | 1,161 | 153 |
|  |  |  |  |  | Staff at a high school in Jerusalem | all | RT-PCR | 2 | 152 | 151 | 25 |

*These studies do not report contacts from index cases but pertain to serological surveys in the school populations in the period of time after schools were open and as such cannot be used to estimate secondary attack rates as it is not clear where these transmission events occurred.

## Workplaces

**Table 3. Summary of studies reporting on contacts of index cases in workplace locations.** Where index cases were not broken down by exposure location the study level number of index cases is provided.

| **Main text reference number** | **Author** | **Region** | **Country** | **Study design** | **Contact definition** | **Testing strategy** | **Testing method** | **Index cases** | **Contacts traced** | **Contacts tested** | **Secondary cases** |
| --- | --- | --- | --- | --- | --- | --- | --- | --- | --- | --- | --- |
| 31 | Chaw et al [18] | Not specified | Brunei | Cluster investigation and contact tracing | Contacts encountered in the workplace | all | RT-PCR | 19 | 848 | 848 | 6 |
| 39 | Chen et al [28] | Ningbo | China | Surveillance and contact tracing | Work | all | RT-PCR | 187 | 47 | 47 | 1 |
|  | Chu et al [64] | Washington | USA | Enhanced contact investigation | Office based workers, with face to face contact inside the office, car-pooling and a shared lunch 2-90 minutes of contact | symptomatic | RT-PCR | 1 | 11 | not specified | 0 |
|  | Guallar et al [34] | Madrid | Spain | Cluster investigation | Adults that met in a small conference room for three hours | unclear | unclear | 1 | 7 | 7 | 7 |
| 41 | Hijnen et al [65] | Munich | Germany | Outbreak investigation and contact tracing | Shared the board room meeting and dinner with the index case | subset | RT-PCR and serology | 1 | 13 | 12 | 11 |
| 40 | Park et al [66] | Seoul | South Korea | Outbreak investigation and contact tracing of a cluster of cases associated with a cluster of cases in a call centre office building | floors 1 – 6 of office and residential building | all | RT-PCR | 2 | 84 | 84 | 0 |
|  |  |  |  |  | call centre floor 7 | all | RT-PCR | 1 | 182 | 182 | 0 |
|  |  |  |  |  | call centre floor 8 | all | RT-PCR | 1 | 207 | 207 | 0 |
|  |  |  |  |  | call centre floor 9 | all | RT-PCR | 1 | 206 | 206 | 1 |
|  |  |  |  |  | call centre floor 10 | all | RT-PCR | 1 | 27 | 27 | 2 |
|  |  |  |  |  | call centre floor 11 | all | RT-PCR | 1 | 216 | 216 | 94 |
| 13 | Pollan et al [46] | Nationwide | Spain | Serosurvey | Workplace contacts | all | Serology | not specified* | 1461 | 1461 | 118 |
| 28 | Zhang et al [61] | Liocheng | China | Outbreak investigation and contact tracing | Employees of a supermarket where a confirmed case worked | all | RT-PCR | 1 | 120 | 120 | 11 |
| 27 | Zhang et al [62] | Guangzhou | China | Surveillance and contact tracing | Co-workers who had contact with index case without using proper protection (surgical mask) during 2 days before the index case was tested | all | RT-PCR | 38 | 119 | 119 | 0 |

* Inferred >1 due to contact sample size

## Healthcare

**Table 4. Summary of studies reporting on contacts of index cases in healthcare locations.** Where index cases were not broken down by exposure location the study level number of index cases is provided.

| **Main text reference number** | **Author** | **Region** | **Country** | **Study design** | **Contact definition** | **Testing strategy** | **Testing method** | **Index cases** | **Contacts traced** | **Contacts tested** | **Secondary cases** |
| --- | --- | --- | --- | --- | --- | --- | --- | --- | --- | --- | --- |
|  | Baker et al [67] | Boston | USA | Retrospective hospital contact investigation | HCWs exposed to the patient during their hospitalisation | subset (symptomatic and optional testing) | RT-PCR | 1 | 44 | 37 | 2 |
| 22 | Burke et al [26] | Multiple | USA | Surveillance and contact tracing | HCWs having contact (>1-2 mins within 6ft) or sharing the same room for >10mins as the case. | subset (symptomatic and high-risk contacts) | RT-PCR | 9 | 163 | 77 | 0 |
|  |  |  |  | Surveillance and contact tracing | Patients having contact (>1-2 mins within 6ft) or sharing the same room for >10mins as the case. | subset (symptomatic and high-risk contacts) | RT-PCR | 9 | 95 | 27 | 0 |
|  | Canova et al [68] | Solothurn | Switzerland | Hospital contact investigation | HCWs exposed to a index case patient without appropriate PPE | all | RT-PCR | 1 | 21 | 21 | 0 |
| 44 | Chen et al [69] | Nanjing | China | Hospital contact investigation | HCWs exposed to a case patient | all | RT-PCR | 4 | 105 | 105 | 18 |
| 39 | Chen et al [28] | Ningbo | China | Surveillance and contact tracing | HCWs providing care to a case | all | RT-PCR | 187 | 72 | 72 | 0 |
|  |  |  |  |  | Patients sharing the same ward as a case | all | RT-PCR | 187 | 225 | 225 | 4 |
| 33 | Cheng et al [29] | Not specified | Taiwan | Surveillance and contact tracing | Medical staff, hospital workers and other patients contacting an index case within 2m without appropriate PPE (N95) | all | RT-PCR | 100 | 698 | 698 | 6 |
|  | Chu et al [64] | Washington | USA | Contact investigation | Healthcare personnel having a face-to-face interaction with an index case or contact with a case patient's secretions without appropriate PPE | symptomatic and subset | RT-PCR | 1 | 8 | not-specified | 0 |
|  |  |  |  |  | Sharing a healthcare waiting room or area during the same time and up to 2 hours after the case-patient was present | symptomatic and subset | RT-PCR | 1 | 31 | not-specified | 0 |
|  | Draper et al [33] | Northern Territory | Australia | Surveillance and contact tracing | HCW with face-to-face contact with a confirmed COVID-19 case for more than 15 minutes cumulatively or continuously (without appropriate use of personal protective equipment) | Symptomatic | RT_PCR | 28 | 4 | not-specified | 0 |
|  | Gao et al [70] | Guandong | China | Contact investigation | Patients exposed to the case | all | RT-PCR | 1 | 35 | 35 | 0 |
|  |  |  |  |  | Healthcare personnel exposed to the case | all | RT-PCR | 1 | 224 | 224 | 0 |
|  | Ghinai et al [71] | Chicago | USA | Contact investigation | HCWs exposed to the patient during hospitalisation and transport to the hospital | symptomatic and subset | RT-PCR | 2 | 195 | 64 | 0 |
|  | Hara et al [72] | Kyoto | Japan | Hospital contact investigation | Patients who were on the ward at the time the HCW index case was working | all | RT-PCR | 1 | 87 | 87 | 1 |
|  | Heinzerling et al [73] | California | USA | Hospital Contact investigation | Healthcare personnel exposed to an index patient identified through medical record review | symptomatic | RT-PCR | 1 | 121 | 43 | 3 |
|  | Korea CDC [74] | Not specified | South Korea | Surveillance and contact tracing | Healthcare personnel exposed to an index patient | symptomatic | RT-PCR | 30 | 233 | unclear | 0 |
|  |  |  |  |  | Patients exposed to an index patient | symptomatic | RT-PCR | 30 | 169 | unclear | 1 |
|  | Laxminaraya et al [39] | Tamil Nadu and Andhra Pradesh | India | Surveillance and contact tracing | Healthcare | subset | RT-PCR | 11 | 210 | not specified | 2 |
| 45 | Liu et al [75] | Guangdong | China | Surveillance and contact tracing | HCW exposure to SARS-CoV-2 positive patients | all | RT-PCR | 1158 | 573 | 573 | 2 |
| 46 | Lombardi et al [76] | Lombardy | Italy | Hospital contact investigation | HCWs who had contact with a patient or another HCW with or later diagnosed SARS-CoV-2 infection | all | RT-PCR | not specified* | 1,573 | 1,573 | 139 |
| 30 | Luo et al [43] | Guangzhou | China | Surveillance and contact tracing | HCWs giving direct care to index patients | all | RT-PCR | 347 | 679 | 679 | 7 |
|  | Ng et al [77] | Simei | Singapore | Hospital contact investigation | HCWs having exposure to aerosol-generating procedures for >10 mins within 2m from the patient | all | RT-PCR | 1 | 41 | 41 | 0 |
| 43 | Pini et al [78] | Emilia-Romagna | Italy | Hospital protocol and contact tracing | Surgeons who had contact with infected colleagues | all | RT-PCR | 2 | 14 | 14 | 0 |
|  | Saban et al [79] | Jerusalem | Israel | Hospital contact investigation | HCWs in contact with the index case for more than 15 mins within 2m | all | RT-PCR | 1 | 11 | 11 | 0 |
|  |  |  |  |  | Patients who were exposed to an ophthalmologist who was a positive case | subset | RT-PCR | 1 | 142 | 16 | 0 |
|  | Schneider [80] | Münster | Germany | Hospital contact investigation | HCW exposed to the patient from day of admission until patient’s final diagnosis were identified using work schedules and the hospital information system | all | RT-PCR | 1 | 66 | 66 | 0 |
|  | Singh et al [81] | Kerala | India | Hospital contact investigation | All hospital contacts of an HCW index case | all | RT-PCR | 1 | 184 | 184 | 0 |
| 42 | Wee et al [82] | ‎Bukit Merah‎ | Singapore | Hospital contact investigation | HCWs who had contact within two metres of the index case patient for a cumulative time of ≥15 minutes, or who had performed AGPs without appropriate PPE | symptomatic | RT-PCR | 5 | 126 | 73 | 0 |
|  |  |  |  |  | Patients sharing the same cubicle as a confirmed patient index case | all | RT-PCR | 5 | 13 | 13 | 1 |
|  | Wee et al [83] | Bukit Merah | Singapore | Hospital contact investigation | HCWs who had contact within two metres of the index case for a cumulative time of ≥15 minutes, or who had performed AGPs without appropriate PPE | symptomatic | RT-PCR | 28 | 253 | 132 | 0 |
|  |  |  |  |  | Patients sharing the same cubicle as a confirmed case | symptomatic | RT-PCR | 28 | 45 | 24 | 1 |
|  | Wee et al [84] | Bukit Merah | Singapore | Hospital contact investigation | HCWs and patients who had contact within two metres of the index case for a cumulative time of ≥15 minutes, or who had performed AGPs without appropriate PPE | symptomatic | RT-PCR | 14 | 148 | 30 | 0 |
|  | Wendt et al [85] | Leipzig | Germany | Hospital contact investigation | HCWs who had interacted with the index patient in the hospital environment | all | RT-PCR and serology | 1 | 187 | 187 | 0 |
|  |  |  |  |  | Patients who had interacted with the index patient in the hospital environment | all | RT-PCR and serology | 1 | 67 | 67 | 0 |
|  | Wong et al [86] | Southern Kowloon | Hong Kong | Hospital contact investigation | Patients who shared the same cubicle with the index | symptomatic | RT-PCR | 1 | 61 | 22 | 0 |
|  |  |  |  |  | Staff who had contact within 2m of the index case for a cumulative time of >15 min, or had performed AGPs, without appropriate PPE | symptomatic | RT-PCR | 1 | 42 | 30 | 0 |
| 21 | Wu et al [56] | Hangzhou | China | Surveillance and contact tracing | A close contact in a medical institution setting, either a healthcare provider or patient, defined as being within 1m of a confirmed case, without effective protection, within 5 days before symptom onset or testing of index case | all | unclear | 144 | 532 | 532 | 2 |
|  | Yu et al [59] | Xinzhou | China | Surveillance and contact tracing | Doctor-patient | unclear | unclear | 560 | 5 | 5 | 2 |
| 26 | Zhang et al [63] | Hunan | China | Surveillance and contact tracing | Healthcare | all | RT-PCR | 136 | 572 | 572 | 7 |

* Inferred >1 due to contact sample size

## Group Living Populations

**Table 5. Summary of studies reporting on contact investigations in group living vulnerable populations**

| **Main text reference number** | **Author** | **Region** | **Country** | **Study design** | **Setting** | **Contact definition** | **Testing strategy** | **Testing method** | **Index cases** | **Contacts traced** | **Contacts tested** | **Secondary cases** |
| --- | --- | --- | --- | --- | --- | --- | --- | --- | --- | --- | --- | --- |
|  | Baggett et al [14] | Boston | USA | Outbreak investigation | Homeless shelter | Adults aged 18+ residing in the shelter on April 2 and April 3, 2020, without being previously diagnosed with COVID-19 | all | RT-PCR | Not stated directly, infered ~16-25 | 408 | 408 | 147 |
|  | Kimball et al [11] | Washington | USA | Outbreak investigation | Care facility | Residents in a skilled nursing facility with a confirmed case | all | RT-PCR | 1 | 76 | 76 | 23 |
|  | Marcus et al [15] | Texas | USA | Surveillance and contact tracing | Military base | Trainees reporting symptoms and either exposure to a known COVID-19 case or travel from a high-transmission area. | symptomatic | RT-PCR | 1 | 4,073 | 86 | 3 |
|  | McMichael et al [13] | Washington | USA | Outbreak investigation | Care facility | Residents living in a skilled nursing with a confirmed case | Subset | RT-PCR | 2 | 170 | 118 | 101 |
|  | Roxby et al [12] | Washington | USA | Outbreak investigation | Care facility | All Residents and healthcare personal of the facility with a confirmed case | all | RT-PCR | 2 | 142 | 142 | 5 |
|  | Wallace et al [16] | Louisiana | USA | Contact investigation | Correctional and detention facilities | Asymptomatic close contacts of incarcerated and detained persons with COVID-19 (Facility A) at the end of their 14-day isolation | all | RT-PCR | Not listed | 10 | 10 | 6 |
|  |  |  |  |  |  | Asymptomatic close contacts of incarcerated and detained persons with COVID-19 at the end of their 14-day isolation (Facility B) | all | RT-PCR | Not listed | 19 | 19 | 9 |

## Social Settings

**Table 6. Summary of studies reporting on contact investigations in different social settings.** Where index cases were not broken down by exposure location the study level number of index cases is provided.

| **Main text reference number** | **Author** | **Region** | **Country** | **Study design** | **Contact definition** | **Testing strategy** | **Testing method** | **Index cases** | **Contacts traced** | **Contacts tested** | **Secondary cases** |
| --- | --- | --- | --- | --- | --- | --- | --- | --- | --- | --- | --- |
| 10 | Bi et al [24] | Shenzhen | China | Surveillance and contact tracing | Travel: Travelled together with the index case 2 days before symptom onset | all | RT-PCR | 391 | 318 | 318 | 18 |
|  |  |  |  |  | Family and Friends: Socially interacted with the index case or shared a meal with an index case 2 days before symptom onset | all | RT-PCR | 391 | 707 | 707 | 61 |
| 11 | Bohmer et al [25] | Bavaria | Germany | Outbreak investigation and contact tracing | Family and Friends: Family and friends having face-to-face contact with confirmed case for at least 15 minutes | all | RT-PCR | 16 | 217 | 217 | 11 |
|  |  |  |  |  | "Low-risk" casual contacts | Symptomatic | RT-PCR | 16 | 108 | 0 | 0 |
| 31 | Chaw et al [18] | Not specified | Brunei | Cluster investigation and contact tracing | Family and Friends: Relatives living outside the household of an index case | all | RT-PCR | 19 | 144 | 144 | 5 |
|  |  |  |  |  | Religious Event: Those who attended a local religious event in Brunei on March 5 | all | RT-PCR | 19 | 54 | 54 | 8 |
|  |  |  |  |  | Casual contacts: Those who encountered an index case during travel or in social events. | all | RT-PCR | 19 | 445 | 445 | 4 |
| 39 | Chen et al [28] | Ningbo | China | Surveillance and contract tracing | Travel: Public transport | all | RT-PCR | 187 | 235 | 235 | 28 |
|  |  |  |  |  | Family and Friends: gathering/ treating guests/ entertainment | all | RT-PCR | 187 | 724 | 724 | 52 |
|  |  |  |  |  | Casual contacts: General population, being in the same building or environment, short talk or task | all | RT-PCR | 187 | 565 | 565 | 10 |
| 33 | Cheng et al [29] | Not specified | Taiwan | Surveillance and contact tracing | Family and Friends: Relatives living outside the household of an index case | Symptomatic | RT-PCR | 100 | 76 | Not specified | 5 |
| 72 | Danis et al [5] | Les Contamines-Montjoie | France | Outbreak investigation and contact tracing | Holiday: Resided in the same chalet as an index case | all | RT-PCR | 1 | 15 | 15 | 11 |
|  |  |  |  |  | Sports: Attended the same ski school as index case | Symptomatic | NA | 1 | 16 | 0 | 0 |
|  |  |  |  |  | Casual contacts: Contacts of an index case including apartment staff and cleaners, staff in shops and restaurants, and passengers in 4 buses and 3 airplanes. | Symptomatic | RT-PCR | 11 | 64 | 12 | 0 |
|  | Draper et al [33] | Northern Territory | Australia | Surveillance and contact tracing | Travel: Aircraft close contacts included passengers seated in the same row as, or in the two rows in front of or behind, an infectious case. If the case was a crew member, the passengers in the area in which the crew member worked were classified as close contacts. | Symptomatic | RT-PCR | 28 | 389 | Not specified | 0 |
|  | Hamner et al [87] | Washington | USA | Outbreak investigation | Religious Event: Attending a choir practice with a confirmed case | Symptomatic | RT-PCR | 1 | 60 | Not specified | 32 |
|  | Huang et al [21] | Anhui | China | Surveillance and contact tracing | Family and Friends: Sharing a dinner/ karaoke/ social get-together with the index case | all | RT-PCR | 1 | 17 | 17 | 7 |
|  | James et al [88] | Arkansas | USA | Contact tracing investigation | Religious Event: Attended a bible study group and other church related events with confirmed cases (pastor and wife) | symptomatic | RT-PCR | 2 | 90 | 45 | 33 |
| 47 | Jang et al [19] | Cheonan | South Korea | Outbreak investigation and contact tracing | Sports: Students of 6 exercise instructors, who were confirmed cases, teaching 12 fitness dance classes | symptomatic | RT-PCR | 6 | 217 | 57 | 57 |
| 37 | Jing et al [38] | Guangzhou | China | Retrospective contact investigation | Family and Friends: family members not living at the same residential address as an index case | all | RT-PCR | 215 | 1,314 | 1314 | 31 |
|  | Laxminaraya et al [39] | Tamil Nadu and Andhra Pradesh | India | Surveillance and contact tracing | Travel: | subset | RT-PCR | 8 | 78 | Not specified | 63 |
|  |  |  |  |  | Combined casual contacts: | subset | RT-PCR | 596 | 9628 | Not specified | 249 |
| 45 | Liu et al [75] | Guangdong | China | Surveillance and contact tracing | Combined casual contacts: social activity contacts | all | RT-PCR | 1158 | 3344 | 3344 | 41 |
|  |  |  |  |  | Travel: flight, train, public transportation, and private car | all | RT-PCR | 1158 | 2038 | 2038 | 31 |
| 30 | Luo et al [43] | Guangzhou | China | Surveillance and contract tracing | Travel: Individuals travelling in the same car, airplane, or train as a confirmed case | all | RT-PCR | 347 | 818 | 818 | 1 |
|  |  |  |  |  | Casual contacts: Other social contacts of a confirmed case | all | RT-PCR | 347 | 875 | 875 | 11 |
| 73 | Pavli et al [17] | | Greece / Israel | Surveillance and contact tracing | Religious Event: Transportation in the same bus/residing in the same hotels/religious rituals/ recreational activities as confirmed cases in a group of Christian pilgrims returning to Greece from Jerusalem. | all | RT-PCR | not specified | 53 | 53 | 48 |
|  |  | |  |  | Travel: Passengers and crew members of the same flight and a bus driver of the tour group | unclear | RT-PCR | not specified | not specified | Not specified | 6 |
| 13 | Pollan et al [46] | Nationwide | Spain | Serosurvey | Family and friends: non-cohabiting family member or friend | all | Serology | not specified* | 1284 | 1284 | 146 |
| 74 | Yusef et al [20] | Irbid | Jordan | Outbreak investigation | Religious Event: Wedding attendees after identification of an index case | all | RT-PCR | 1 | 350 | 350 | 76 |
| 17 | Wang et al [54] | Beijing | China | Surveillance and contact tracing | Casual contacts: social contacts | all | RT-PCR | 602 | 3363 | 3363 | 75 |
| 26 | Zhang et al [63] | Hunan | China | Surveillance and contact tracing | Travel: Travel associated contacts of a confirmed case | all | RT-PCR | 136 | 326 | 326 | 22 |
| 27 | Zhang et al [62] | Guangzhou | China | Surveillance and contact tracing | Family and Friends: Visiting or sharing meals with a confirmed case without using proper protection (surgical mask) during 2 days before the index case was tested. | all | RT-PCR | 38 | 66 | 66 | 1 |
|  |  |  |  |  | Casual contacts: Providing catering and entertainment services in a closed environment, sharing transportation, such as in vehicle, or on a flight or ship without using proper protection (surgical mask) during 2 days before the index case was tested. | all | RT-PCR | 38 | 122 | 122 | 1 |
| 28 | Zhang et al [61] | Liocheng | China | Outbreak investigation and contact tracing | Casual contacts: customers using the supermarket during the days the index cases were identified inside the supermarket | all | RT-PCR | 12 | 8,224 | 8,224 | 2 |

* Inferred >1 due to contact sample size

## Combined close contacts

**Table 7 Summary of studies reporting on contact investigations where contacts of index cases were not disaggregated by exposure location**

| **Main text reference number** | **Author** | **Region** | **Country** | **Study design** | **Contact definition** | **Testing strategy** | **Testing method** | **Index cases** | **Contacts traced** | **Contacts tested** | **Secondary cases** |
| --- | --- | --- | --- | --- | --- | --- | --- | --- | --- | --- | --- |
|  | Aghaali et al [89] | Qom | Iran | Surveillance and contact tracing | Contact with a confirmed case (<2m away) during symptomatic period, including 4 days before symptom onset (defined as close contact). | symptomatic | X-rays and chest CT | 51 | 318 | Not specified | 37 |
|  | Barrett et al [90] | Cork/Kerry | Ireland | Surveillance and contact tracing | casual (< 15 min face-to-face exposure) or close (≥ 15 min face-to-face exposure) contacts of confirmed cases | symptomatic | RT-PCR | not stated | 1,336 | 120 | 35 |
| 22 | Burke et al [26] | Multiple | USA | Surveillance and contact tracing | Combined close contacts: flight-related contacts (passengers seated in the same or within one row of the index case and airport quarantine station screeners), rideshare drivers, and friends/community contacts. | subset (symptomatic and high-risk contacts) | RT-PCR | 9 | 65 | 44 | 0 |
| 33 | Cheng et al [29] | Not specified | Taiwan | Surveillance and contact tracing | Friends, airline crew members and passengers and other casual contacts of index cases | symptomatic | RT-PCR | 100 | 1,836 | Not specified | 1 |
|  | Ge et al [91] | Jiaxing | China | Surveillance and contact tracing | People who had unprotected close contact with a case were defined as close contacts, such as those living, studying, or working together, or those travelling on the same vehicle during onset | symptomatic | RT-PCR | 1 | 547 | Not specified | 6 |
| 49 | Han et al [92] | Hangzhou | China | Contact investigation of asymptomatic cases | Close contacts of asymptomatic confirmed cases | all | RT-PCR | 18 | 41 | 41 | 0 |
|  | Heavey et al [8] | Not specified | Ireland | Surveillance and contact tracing | Households of friends and family and recreational activities with index cases | unclear | RT-PCR | 6 | 135 | NA | 2 |
| 50 | Hong et al [93] | Taizhou, Zhejiang | China | Surveillance and contact tracing | Close contacts including family members, relatives, friends, villagers, or card playing partners of pre-symptomatic index cases who wore a mask | all | RT-PCR | 41 | 123 | 123 | 10 |
|  |  |  |  |  | Close contacts including family members, relatives, friends, villagers, or card playing partners of pre-symptomatic index cases who did not wear a mask | all | RT-PCR | 41 | 74 | 74 | 14 |
|  | Jia et al [94] | Qingdao | China | Surveillance and contact investigation | Close contacts who did not take effective protection against a suspected or confirmed case 2 days before symptom onset or sampling. Contacts either lived with, shared a meal with an index case, or came in contact with an index case in a healthcare setting. | all | RT-PCR | 11 | 583 | 583 | 33 |
| 51 | Jiang et al [37] | Shandong Province | China | Surveillance and contact tracing | Contact with confirmed cases within 1m without wearing proper personal protection | all | RT-PCR | 7 | 300 | 300 | 6 |
| 48 | Kwok et al [95] | Not specified | Hong Kong | Surveillance and contact tracing | Close contacts referred to anyone who: (i) provided care to the case (including family or HCW) or had other close physical contact; or (ii) stayed at the same place (including household members or visitors) while the case was ill | unclear | RT-PCR | 27 | 206 | Not specified | 24 |
| 523 | Liu et al [96] | Anhui | China | Contact investigation of asymptomatic cases | Close contacts of asymptomatic confirmed cases | all | RT-PCR | 147 | 1,150 | 1,150 | 47 |
|  | Liu et al [97] | Shiyan City | China | Surveillance and contact tracing | Family members living in the same room, medical workers without secondary protection, and sharing personal meals or communication in confined spaces within 14 days before onset | all | RT-PCR | 176 | 689 | 689 | 85 |
| 30 | Luo et al [43] | Guangzhou | China | Surveillance and contact tracing | Multiple modes of contact with an index case | all | RT-PCR | 347 | 92 | 92 | 12 |
| 12 | Park et al [45] |  | South Korea | Surveillance and contact tracing | Non-household close contacts | symptomatic | RT-PCR | 5706 | 48,481 | Unclear | 921 |
|  | Valent et al [98] | Friuli-Venezia Giulia | Italy | Surveillance and contact tracing | All close contacts of an index case | Subset | RT-PCR | 18 | 142 | 79 | 18 |
|  | Wong et al [99] | Not specified | Brunei | Contact tracing investigation of cases who retested positive | A close contact, defined as living in the same household, or within 1m of a confirmed case in an enclosed space for >15 minutes | all | RT-PCR | 21 | 111 | 111 | 1 |
| 21 | Wu et al [56] | Hangzhou | China | Retrospective contact tracing investigation | A close contact (within 1m of a confirmed case, without effective protection, within 5 days before symptom onset or sampling date of index case) occurring in a public place, workplace, education institution or place of entertainment | all | RT-PCR | 144 | 1,115 | 1,115 | 19 |
|  | Yu et al [59] | Xinzhou | China | Surveillance and contact tracing | Close contacts defined as those who shared meals, travelled, or had social interactions with a confirmed case two days before symptom onset | unclear | unclear | 560 | 170 | Unclear | 3 |
| 54 | Zhang et al [100] | Yancheng | China | Surveillance and contact tracing | Close contacts of an index case | all | RT-PCR and serology | 14 | 274 | 274 | 10 |
| 26 | Zhang et al [63] | Hunan | China | Surveillance and contact tracing | Other contacts of a confirmed case - other refers to non-household, non-healthcare, and nontravel related | all | RT-PCR | 136 | 5521 | 5521 | 292 |

| **Study** | **Number of Index Cases** | **Number of Secondary cases** | **R_obs_** |
| --- | --- | --- | --- |
| Mahapure et al [44] | 4 | 22 | 5.50 |
| Burke et al [26] | 8 | 2 | 0.25 |
| Zhang et al [61] | 13 | 12 | 0.92 |
| Bohmer et al [25] | 16 | 5 | 0.31 |
| Chaw et al [18] | 19 | 16 | 0.84 |
| Dong et al [32] | 26 | 53 | 2.04 |
| Dawson et al [31] | 26 | 16 | 0.62 |
| Xin et al [35] | 31 | 19 | 0.61 |
| Wu et al [55] | 35 | 48 | 1.37 |
| Zhang et al [62] | 38 | 10 | 0.26 |
| van der Hoek et al [50] | 54 | 49 | 0.91 |
| Li et al [40] | 105 | 64 | 0.61 |
| Wang et al [52] | 124 | 77 | 0.62 |
| Sun et al [49] | 148 | 189 | 1.28 |
| Rosenberg et al [48] | 155 | 131 | 0.85 |
| Jing et al [38] | 215 | 103 | 0.48 |
| Yung et al [60] | 223 | 13 | 0.06 |
| Dattner et al [30] | 637 | 873 | 1.37 |
| Park et al [45] | 5706 | 1248 | 0.22 |
| **Summary** | | | **0.96**  **(95% CI: 0.67-1.32)** |

**Table 8 Observed and Poisson-Gamma model estimated R_obs_ across household settings.** Studies were included in the pooling where it was clear the number of index cases listed all had household contacts

| **Study** | **Number of Index Cases** | **Number of Secondary cases** | **R_obs_** |
| --- | --- | --- | --- |
| **Up to 5 days** | | | |
| Burke et al [26] | 6 | 0 | 0.0 |
| Bohmer et al [25] | 15 | 2 | 0.13 |
| Chaw et al [18] | 16 | 14 | 0.88 |
| Li et al [40] | 46 | 24 | 0.52 |
| **Summary** | | | **0.40**  **(95% CI: 0.21-0.72)** |
| **5 days or more** | | | |
| Bohmer et al [25] | 1 | 3 | 3.00 |
| Burke et al [26] | 2 | 2 | 1.00 |
| Chaw et al [18] | 3 | 2 | 0.67 |
| Mahapure et al [44] | 4 | 22 | 5.50 |
| Li et al [40] | 59 | 40 | 0.68 |
| **Summary** | | | **1.91**  **(95% CI: 0.86-3.55)** |
| **Summary (excluding Mahapure et al)*** | | | **1.05**  **(95% CI: 0.32-2.26)** |

**Table 9 Observed and Poisson-Gamma model estimated R_obs_ across household settings where contacts experienced different durations of exposure to symptomatic index cases.** Studies were included only where the number of index cases, their contacts and secondary cases were stratified by exposure duration.

* This ≤5 days estimate was sensitive to the inclusion of a familial outbreak from a large dwelling in India whose exclusion resulted in an R_obs_ of1.05 (95% CI: 0.32-2.26).

**Table 10 Observed and Poisson-Gamma estimated R_obs_ across healthcare settings.** Studies were included in the pooling where it was clear the number of index cases listed all had healthcare contacts.

| **Study** | **Number of Index Cases** | **Number of Secondary cases** | **R_obs_** |
| --- | --- | --- | --- |
| Burke et al [26] | 2 | 0 | 0.00 |
| Bohmer et al [25] | 4 | 18 | 4.50 |
| Wee et al [82] | 5 | 1 | 0.20 |
| Li et al [40] | 100 | 6 | 0.06 |
| **Summary** | | | **1.17**  **(95% CI: 0.65-2.04)** |

| **Study** | **Number of Index Cases** | **Number of Secondary cases** | **R_obs_** |
| --- | --- | --- | --- |
| Bohmer et al [25] | 16 | 11 | 0.69 |
| Chaw et al [18] | 19 | 5 | 0.26 |
| Jing et al [38] | 215 | 31 | 0.14 |
| **Summary** | | | **0.38**  **(95% CI: 0.01-0.64)** |

**Table 11 Observed and Poisson-Gamma estimated R_obs_ across family and friend contacts. Only three** studies were included in the pooling where it was clear the number of index cases listed all had family and friend contacts.

| **Study** | **Number of Index Cases** | **Number of Secondary cases** | **R_obs_** |
| --- | --- | --- | --- |
| **Index case aged 0-19 years** | | | |
| Chaw et al [18] | 1 | 1 | 1.00 |
| Van der Hoek et al [50] | 10 | 0 | 0.00 |
| Bi et al [24] | 14 | 2 | 0.14 |
| **Summary** | | | **0.39**  **(95% CI: 0.02-1.35)** |
| **Index case aged 20+ years** | | | |
| Burke et al [26] | 9 | 2 | 0.22 |
| Chaw et al [18] | 18 | 50 | 2.77 |
| Kwok et al [95] | 27 | 24 | 0.89 |
| Bi et al [24] | 220 | 85 | 0.39 |
| Van der Hoek et al [50] | 221 | 55 | 0.25 |
| Yung et al [60] | 223 | 13 | 0.06 |
| **Summary** | | | **0.76**  **(95% CI: 0.52-1.06)** |

**Table 12 Observed and Poisson-Gamma model estimated R_obs_ from child and adult index cases.** Studies were included only when the number of index cases per age category were provided along with their corresponding contacts and secondary cases (across all exposure locations).

| Author | Study Title | Definitions and Duration of follow up |
| --- | --- | --- |
| Chaw et al [18] | Analysis of SARS-CoV-2 Transmission in Different Settings, Brunei | Asymptomatic cases had no symptoms at the time of swab collection or during admission to the national isolation centre – to be discharged required two negative tests in 24 hours. |
| Chen et al [28] | Epidemiological characteristics of infection of close contacts of new coronavirus pneumonia in Ningbo | Diagnosis of asymptomatic cases in line with the national coronavirus prevention and control plan which states that the infected person has a positive nucleic acid test, and after a 14-day incubation period, there is no self-perceived or clinically identifiable symptoms and signs, and the infection is always asymptomatic. |
| Han et al [92] | Severe Acute Respiratory Syndrome Coronavirus 2 among Asymptomatic Workers Screened for Work Resumption, China | In the text of this paper, it quotes “none of the 18 asymptomatic persons in our study developed symptoms” with each case followed on average for 19 days since test positive – ranged from 3 – 41 days. Two of 18 index cases had follow up duration of <14 days. A sensitivity analysis removing this study had no significant impact on the pooled result (pooled result with the study excluded: SAR 2.0% [95% CI: 0.5%-3.4%]). |
| Jiang et al [37] | Transmission Potential of Asymptomatic and Paucisymptomatic Severe Acute Respiratory Syndrome Coronavirus 2 Infections: A 3-Family Cluster Study in China | Each asymptomatic patient had 16-25 days follow up from positive test and remained asymptomatic throughout. |
| Liu et al [96] | The assessment of transmission efficiency and latent infection period on asymptomatic carriers of SARS-CoV-2 infection | All asymptomatic carriers fulfilled the following criteria: (1) without symptoms of fever, cough, and fatigue; (2) no radiographic evidence of pneumonia;(3) with normal white cell count and normal lymphocyte count;  and (4) positive nucleic acid test for SARS-CoV-2.  Study had a 14 days observation period with those not developing symptoms during this period classified as asymptomatic. |
| Luo et al [43] | Modes of contact and risk of transmission in COVID-19 among close contacts | Asymptomatic infection must have not clinical symptoms, must be positive for the virus’ nucleic-acid, and have or be free of radiological and/or laboratory alterations that  indicate viral infection. All confirmed cases were followed up for at least 14 days. |
| Park et al [66] | Coronavirus disease outbreak in call center, South Korea | An asymptomatic case-patients as confirmed a case-patient with a positive COVID-19 test result who remained asymptomatic during the entire 14-day period |
| Zhang et al [62] | Secondary Transmission of Coronavirus Disease from Presymptomatic Persons, China | Asymptomatic person has a positive nucleic acid test, but after the 14-day incubation period the  person remains asymptomatic; no self-perceived or clinically recognized symptoms or signs ever  manifest. |

**Table 13 Asymptomatic case definitions and follow up durations of studies reporting on asymptomatic index cases.**

**Table 14 Observed and Poisson-Gamma model estimated R_obs_ from index cases with different symptom statuses.** Studies were only included when the number of index cases per category was listed along with their contacts and secondary cases.

| **Study** | **Number of Index Cases** | | **Number of Secondary cases** | | | **R_obs_** |
| --- | --- | --- | --- | --- | --- | --- |
| **Asymptomatic index case** | | | | | | |
| Jiang et al [37] | 3 | | 1 | | | 0.33 |
| Chaw et al [18] | 4 | | 2 | | | 0.50 |
| Park et al [66] | 4 | | 0 | | | 0.00 |
| Zhang et al [62] | 12 | | 1 | | | 0.08 |
| Han et al [92] | 18 | | 0 | | | 0.00 |
| Chen et al [28] | 30 | | 6 | | | 0.20 |
| Liu et al [96] | 131 | | 24 | | | 0.18 |
| **Summary** | | | | | | **0.17**  **(95% CI: 0.04-0.45)** |
| **Pre-symptomatic index case** | | | | | | |
| Jiang et al [37] | 3 | | 4 | | | 1.33 |
| Park et al [66] | 4 | | . | | | 0.00 |
| Jang et al [19]* | 6 | | 57 | | | 9.50 |
| Chaw et al [18] | 7 | | 4 | | | 0.57 |
| Hong et al [93] | 13 | | 14 | | | 1.07 |
| Liu et al [96] | 16 | | 23 | | | 1.44 |
| Zhang et al [62] | 71 | | 11 | | | 0.16 |
| **Summary** | | | | | | **1.95**  **(95% CI: 1.28-2.87)** |
| **Summary (excluding Jang et al)*** | | | | | | **0.78**  **(95% CI: 0.36-1.44)** |
| **Symptomatic index case** | | | | | | |
| Jiang et al [37] | | 2 | | 2 | 1.00 | |
| Chen et al [69] | | 4 | | 18 | 4.50 | |
| Chaw et al [18] | | 8 | | 10 | 1.25 | |
| Burke et al [26] | | 9 | | 2 | 0.22 | |
| Wang et al [51] | | 25 | | 10 | 0.0 | |
| Park et al [66] | | 89 | | 34 | 0.38 | |
| Li et al [40] | | 105 | | 64 | 0.61 | |
| Wang et al [52] | | 124 | | 77 | 0.62 | |
| Chen et al [28] | | 157 | | 126 | 0.80 | |
| **Summary** | | | | | | **1.01**  **(95% CI: 0.57-1.61)** |

* The R_obs_ from pre-symptomatic index cases was highly sensitive to the inclusion of a single study from a cluster outbreak related to fitness instructors at sports complex in South Korea.

##
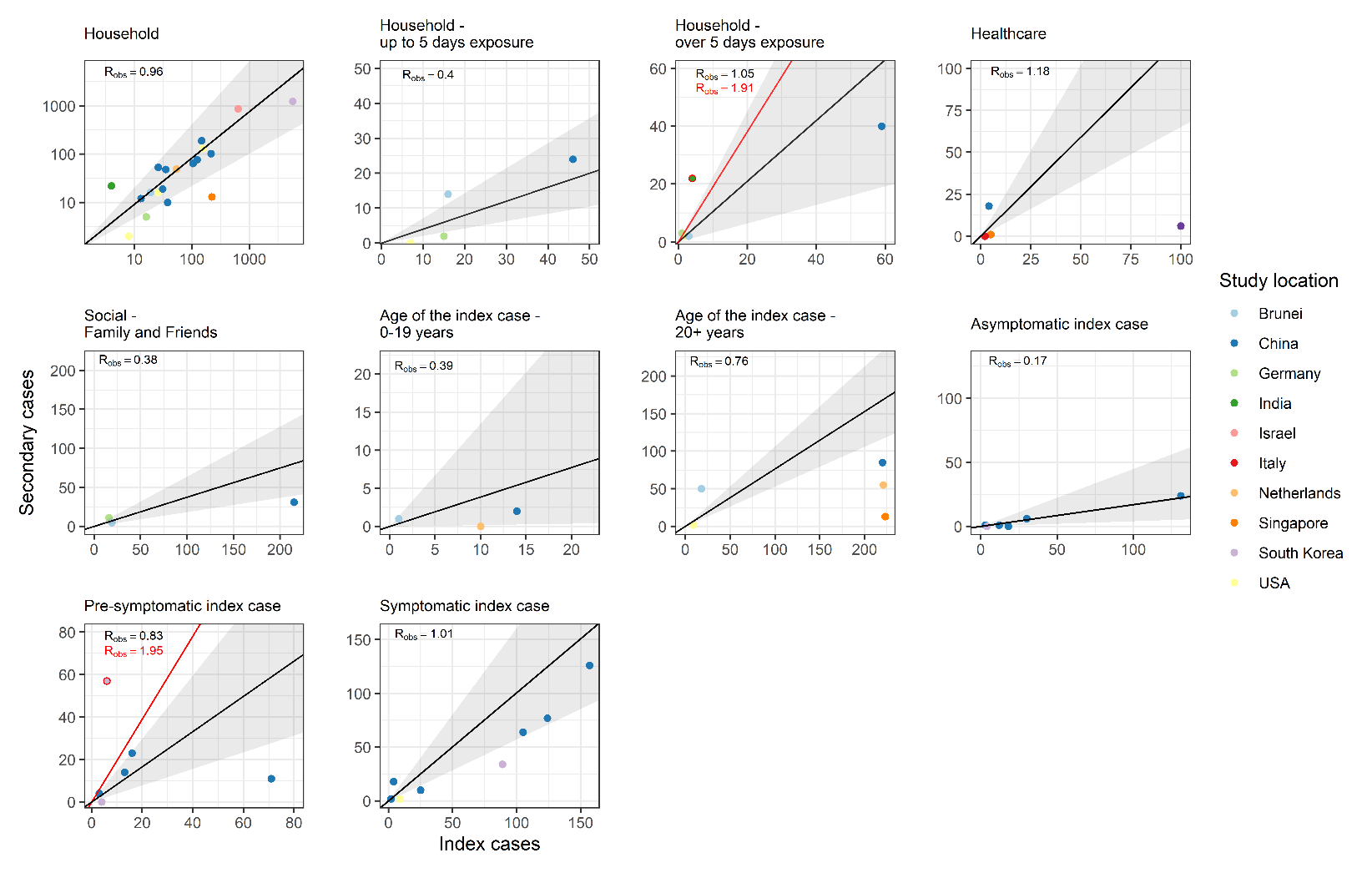
Figures

**Figure 1. Relationship between the number of index cases per study and the observed number of secondary cases.** Black line represents the expected number of secondary cases for observed numbers of index cases according to the model fitted R_obs_ across studies and the shaded area the 95% Confidence Interval of this estimate. Points circled in red represent studies whose inclusion resulted in the R_obs_ shown in red line. Studies are shown only where it was clear that all index cases had setting specific contacts and thus the potential to transmit in this environment or to the contact group listed. Household only plot is drawn with a log10 axis transformation.


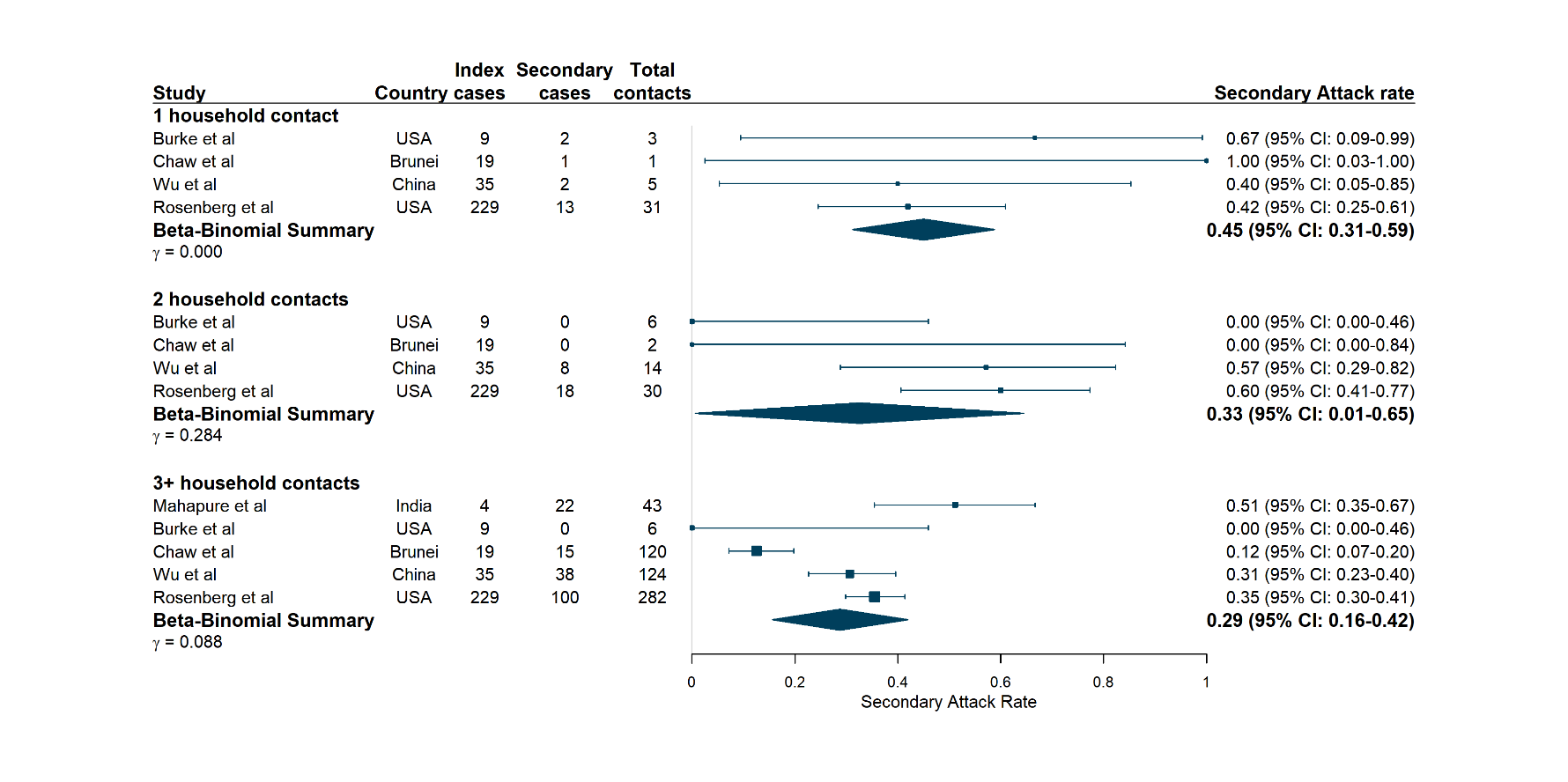
**Figure 2. Estimates of secondary attack rates stratified by household size.** Household size defined as the number of contacts in the household excluding the index case. Studies are ordered by the number of index cases reported in the study as this was not provided consistently at the household size level. There was no significant difference in group level estimates of SAR, p-value = 0.10.


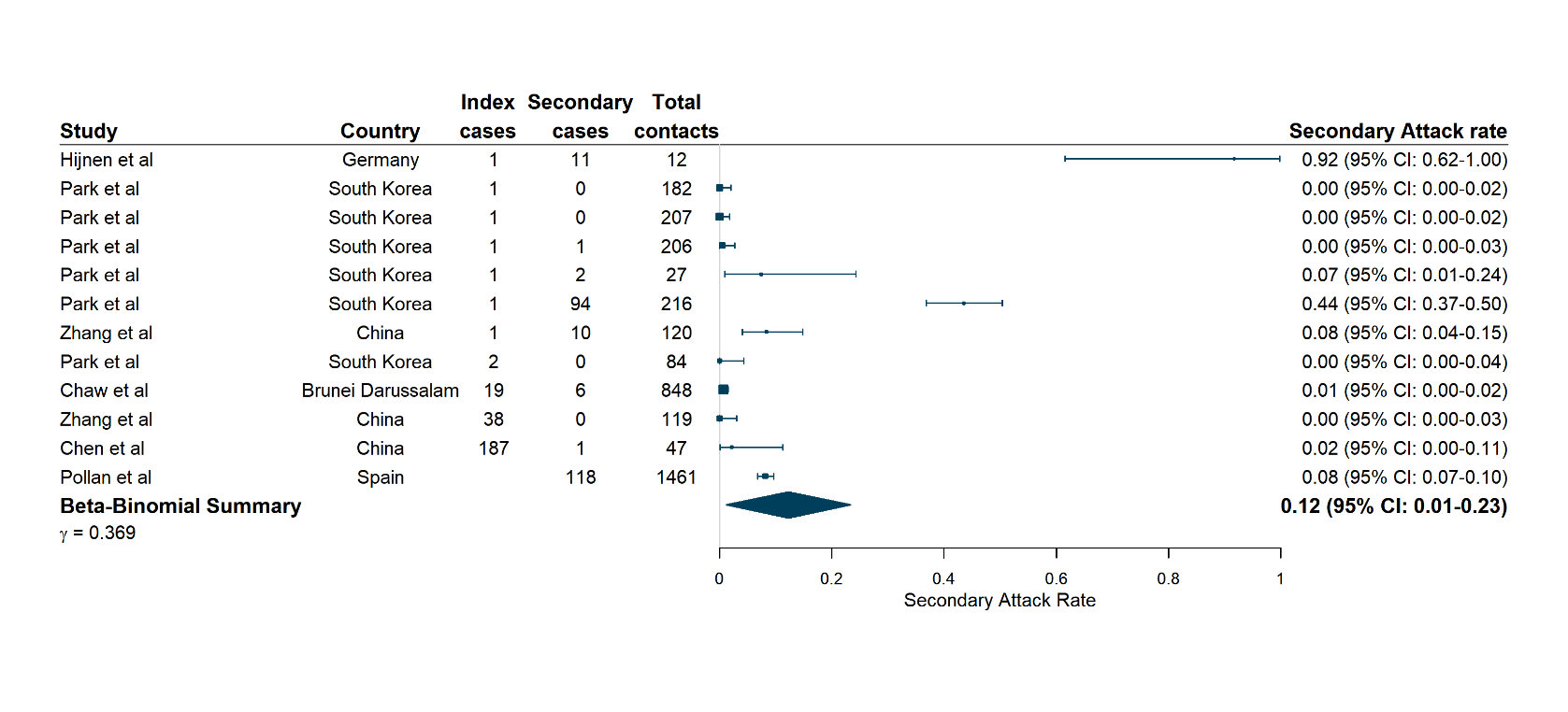
**Figure 3. Estimates of secondary attack rates in workplace contacts including the studies reporting on outbreak clusters from a single index case.** Studies are ordered by the number of index cases reported in the study. Where the number of index cases are missing these were not reported in the study but represent large contact tracing investigations.


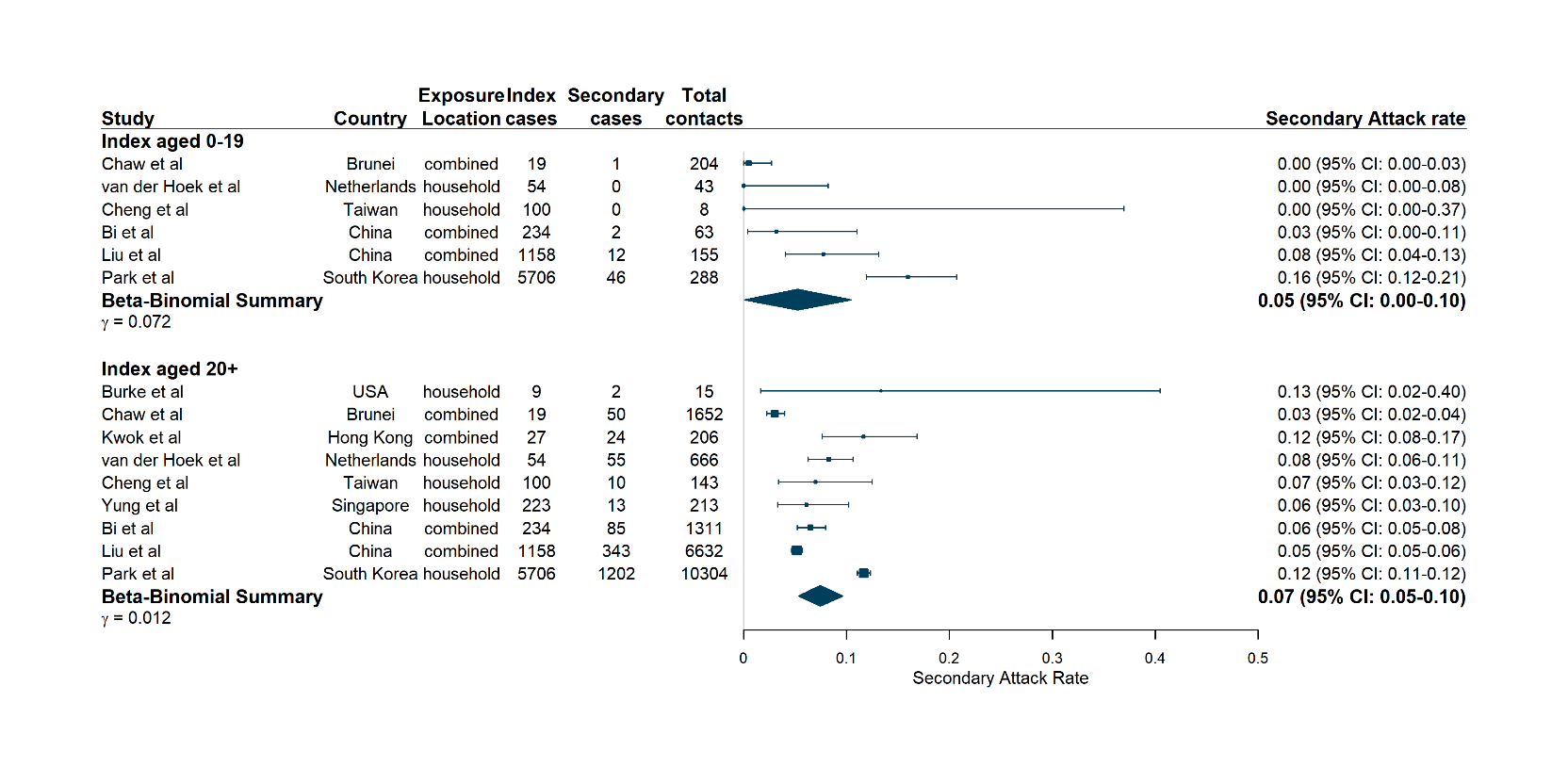


**Figure 4. Estimates of secondary attack rates by age of the index case.** Index age stratified by those aged 0-19 years and those 20+ years, p-value = 0.12. Studies are ordered by the number of index cases reported in the study.

**
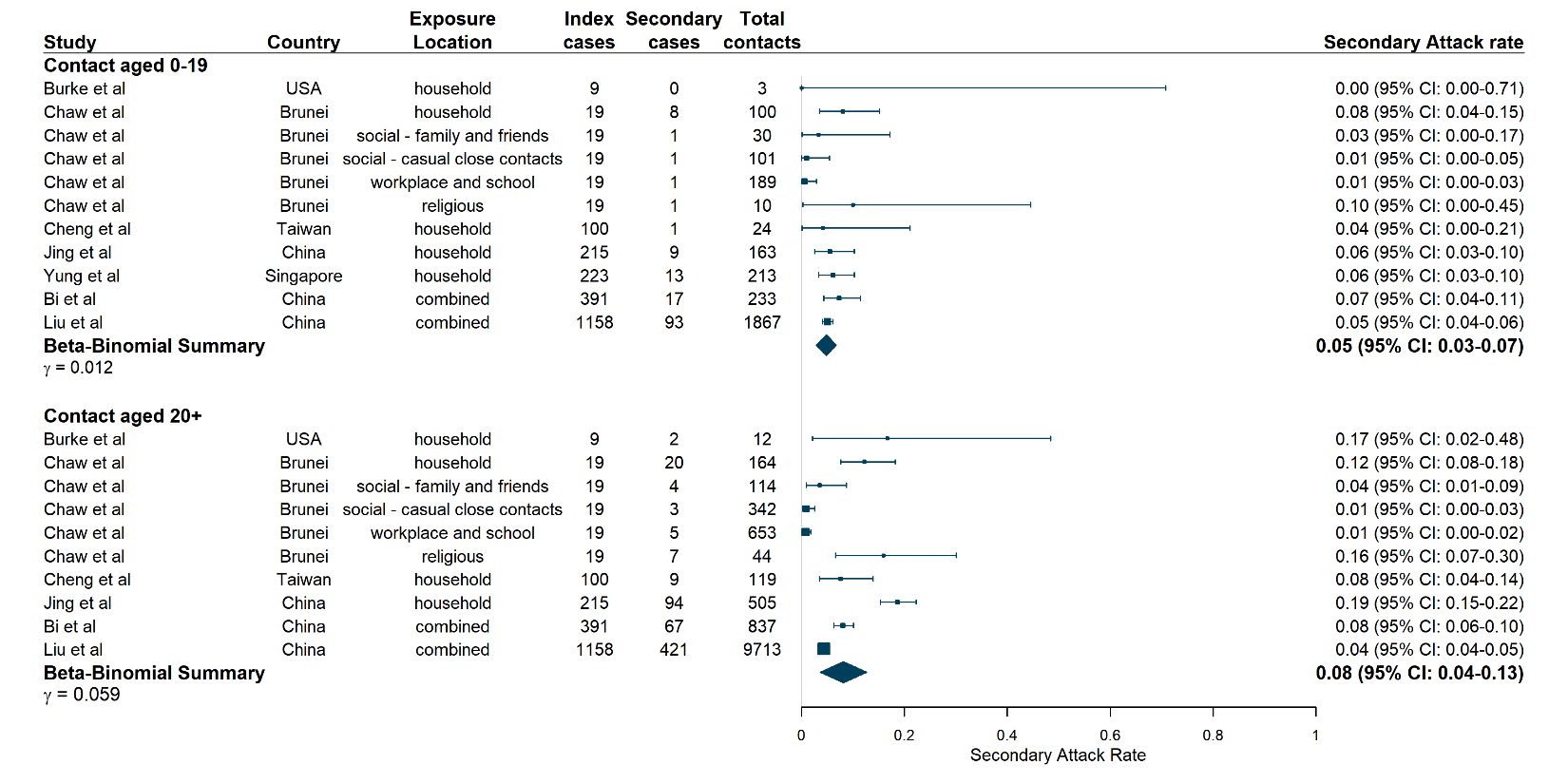
Figure 5. Estimates of secondary attack rates by age of contacts.** Contact age stratified by those aged 0-19 years and those 20+ years, p-value = 0.43. Studies are ordered by the number of index cases reported in the study.

## References

1. Williams DA. 394: The Analysis of Binary Responses from Toxicological Experiments Involving Reproduction and Teratogenicity. Biometrics **1975**; 31:949.

2. Pham T V., Piersma SR, Warmoes M, Jimenez CR. On the beta-binomial model for analysis of spectral count data in label-free tandem mass spectrometry-based proteomics. Bioinformatics **2009**; 26:363–369.

3. Cai T, Parast L, Ryan L. Meta-analysis for rare events. Stat Med **2010**; 29:2078–2089. Available at: /pmc/articles/PMC2932857/?report=abstract. Accessed 19 November 2020.

4. Baettig SJ, Parini A, Cardona I, Morand GB. Case series of coronavirus (SARS-CoV-2) in a military recruit school: clinical, sanitary and logistical implications. BMJ Mil Heal **2020**;

5. Danis K, Epaulard O, Bénet T, et al. Cluster of Coronavirus Disease 2019 (COVID-19) in the French Alps, February 2020. Clin Infect Dis **2020**; 71:825–832. Available at: https://doi.org/10.1093/cid/ciaa424.

6. Fontanet A, Tondeur L, Madec Y, et al. Cluster of COVID-19 in northern France: A retrospective closed cohort study. medRxiv **2020**; :2020.04.18.20071134. Available at: https://www.medrxiv.org/content/medrxiv/early/2020/04/23/2020.04.18.20071134.full.pdf.

7. Fontanet A, Grant R, Tondeur L, et al. SARS-CoV-2 infection in primary schools in northern France: A retrospective cohort study in an area of high transmission. medRxiv **2020**; :2020.06.25.20140178. Available at: https://www.medrxiv.org/content/medrxiv/early/2020/06/29/2020.06.25.20140178.1.full.pdf.

8. Heavey L, Casey G, Kelly C, Kelly D, McDarby G. No evidence of secondary transmission of COVID-19 from children attending school in Ireland, 2020. Euro Surveill **2020**; 25:2000903. Available at: https://pubmed.ncbi.nlm.nih.gov/32489179.

9. Macartney K, Quinn HE, Pillsbury AJ, et al. Transmission of SARS-CoV-2 in Australian educational settings: a prospective cohort study. Lancet Child Adolesc Heal **2020**;

10. Stein-Zamir C, Abramson N, Shoob H, et al. A large COVID-19 outbreak in a high school 10 days after schools’ reopening, Israel, May 2020. Euro Surveill **2020**; 25:2001352. Available at: https://pubmed.ncbi.nlm.nih.gov/32720636.

11. Kimball A, Hatfield KM, Arons M, et al. Asymptomatic and presymptomatic SARS-CoV-2 infections in residents of a long-term care skilled nursing facility—King County, Washington, March 2020. Morb Mortal Wkly Rep **2020**; 69:377.

12. Roxby AC, Greninger AL, Hatfield KM, et al. Detection of SARS-CoV-2 among residents and staff members of an independent and assisted living community for older adults—Seattle, Washington, 2020. Morb Mortal Wkly Rep **2020**; 69:416.

13. McMichael TM, Currie DW, Clark S, et al. Epidemiology of Covid-19 in a Long-Term Care Facility in King County, Washington. N Engl J Med **2020**; 382:2005–2011.

14. Baggett TP, Keyes H, Sporn N, Gaeta JM. Prevalence of SARS-CoV-2 Infection in Residents of a Large Homeless Shelter in Boston. JAMA **2020**; 323:2191–2192. Available at: https://doi.org/10.1001/jama.2020.6887.

15. Marcus JE, Frankel DN, Pawlak MT, et al. COVID-19 Monitoring and Response Among U.S. Air Force Basic Military Trainees - Texas, March-April 2020. MMWR Morb Mortal Wkly Rep **2020**; 69:685–688.

16. Wallace M. Public Health Response to COVID-19 Cases in Correctional and Detention Facilities—Louisiana, March–April 2020. MMWR Morb Mortal Wkly Rep **2020**; 69.

17. Pavli A, Smeti P, Papadima K, et al. A cluster of COVID-19 in pilgrims to Israel. J Travel Med **2020**; Available at: https://doi.org/10.1093/jtm/taaa102.

18. Chaw L, Koh WC, Jamaludin SA, Naing L, Alikhan MF, Wong J. Analysis of SARS-CoV-2 Transmission in Different Settings, Brunei. Emerg Infect Dis J **2020**; 26. Available at: https://wwwnc.cdc.gov/eid/article/26/11/20-2263_article.

19. Jang S, Han SH, Rhee JY. Cluster of Coronavirus Disease Associated with Fitness Dance Classes, South Korea. Emerg Infect Dis **2020**; 26:1917–1920.

20. Yusef D, Hayajneh W, Awad S, et al. Large Outbreak of Coronavirus Disease among Wedding Attendees, Jordan. Emerg Infect Dis **2020**; 26.

21. Huang L, Zhang X, Zhang X, et al. Rapid asymptomatic transmission of COVID-19 during the incubation period demonstrating strong infectivity in a cluster of youngsters aged 16-23 years outside Wuhan and characteristics of young patients with COVID-19: A prospective contact-tracing study. J Infect **2020**; 80:e1–e13.

22. Arnedo-Pena A, Sabater-Vidal S, Meseguer-Ferrer N, et al. COVID-19 secondary attack rate and risk factors in household contacts in Castellon (Spain): Preliminary report. Rev Enf Emerg **2020**; 19:64–70.

23. Bai SL, Wang JY, Zhou YQ, et al. [Analysis of the first cluster of cases in a family of novel coronavirus pneumonia in Gansu Province]. Zhonghua Yu Fang Yi Xue Za Zhi **2020**; 54:E005.

24. Bi Q, Wu Y, Mei S, et al. Epidemiology and transmission of COVID-19 in 391 cases and 1286 of their close contacts in Shenzhen, China: a retrospective cohort study. Lancet Infect Dis **2020**; 20:911–919. Available at: https://pubmed.ncbi.nlm.nih.gov/32353347.

25. Böhmer MM, Buchholz U, Corman VM, et al. Investigation of a COVID-19 outbreak in Germany resulting from a single travel-associated primary case: a case series. Lancet Infect Dis **2020**; 20:920–928. Available at: https://pubmed.ncbi.nlm.nih.gov/32422201.

26. Burke RM, Balter S, Barnes E, et al. Enhanced contact investigations for nine early travel-related cases of SARS-CoV-2 in the United States. PLoS One **2020**; 15.

27. Chen M, Fan P, Liu Z, et al. A SARS-CoV-2 familial cluster infection reveals asymptomatic transmission to children. J Infect Public Health **2020**; 13:883–886. Available at: http://www.sciencedirect.com/science/article/pii/S1876034120304846.

28. Chen Y, Wang A, Yi B, et al. Epidemiological analysis of infection among close contacts of novel coronavirus pneumonia in Ningbo. Chinese J Epidemiol **2020**; 41.

29. Cheng H-Y, Jian S-W, Liu D-P, et al. Contact Tracing Assessment of COVID-19 Transmission Dynamics in Taiwan and Risk at Different Exposure Periods Before and After Symptom Onset. JAMA Intern Med **2020**; Available at: https://doi.org/10.1001/jamainternmed.2020.2020.

30. Dattner I, Goldberg Y, Katriel G, et al. The role of children in the spread of COVID-19: Using household data from Bnei Brak, Israel, to estimate the relative susceptibility and infectivity of children. medRxiv **2020**;

31. Dawson P, Rabold EM, Laws RL, et al. Loss of Taste and Smell as Distinguishing Symptoms of Coronavirus Disease 2019. Clin Infect Dis **2020**; Available at: https://doi.org/10.1093/cid/ciaa799.

32. Dong XC, Li JM, Bai JY, et al. [Epidemiological characteristics of confirmed COVID-19 cases in Tianjin]. Zhonghua Liu Xing Bing Xue Za Zhi **2020**; 41:638–641. Available at: http://europepmc.org/abstract/MED/32164400.

33. Draper AD, Dempsey KE, Boyd RH, et al. The first 2 months of COVID-19 contact tracing in the Northern Territory of Australia, March-April 2020. Commun Dis Intell **2020**; 44. Available at: https://pubmed.ncbi.nlm.nih.gov/32615916/. Accessed 21 October 2020.

34. Guallar MP, Meiriño R, Donat-Vargas C, Corral O, Jouvé N, Soriano V. Inoculum at the time of SARS-CoV-2 exposure and risk of disease severity. Int J Infect Dis **2020**; 97:290–292. Available at: http://www.sciencedirect.com/science/article/pii/S1201971220304707.

35. Hu Z, Song C, Xu C, et al. Clinical characteristics of 24 asymptomatic infections with COVID-19 screened among close contacts in Nanjing, China. Sci China Life Sci **2020**; 63:706–711.

36. Hua C, Miao Z, Zheng J, et al. Epidemiological features and viral shedding in children with SARS‐CoV‐2 infection. J Med Virol **2020**;

37. Jiang XL, Zhang XL, Zhao XN, et al. Transmission Potential of Asymptomatic and Paucisymptomatic Severe Acute Respiratory Syndrome Coronavirus 2 Infections: A 3-Family Cluster Study in China. J Infect Dis **2020**; 221:1948–1952.

38. Jing Q-L, Liu M-J, Zhang Z-B, et al. Household secondary attack rate of COVID-19 and associated determinants in Guangzhou, China: a retrospective cohort study. Lancet Infect Dis **2020**;

39. Laxminarayan R, Wahl B, Dudala SR, et al. Epidemiology and transmission dynamics of COVID-19 in two Indian states. Science (80- ) **2020**;

40. Li W, Zhang B, Lu J, et al. Characteristics of Household Transmission of COVID-19. Clin Infect Dis **2020**; Available at: https://doi.org/10.1093/cid/ciaa450.

41. Li P, Fu J-B, Li K-F, et al. Transmission of COVID-19 in the terminal stages of the incubation period: A familial cluster. Int J Infect Dis **2020**; 96:452–453. Available at: http://www.sciencedirect.com/science/article/pii/S1201971220301466.

42. Li J, Gong X, Wang Z, et al. Clinical features of familial clustering in patients infected with 2019 novel coronavirus in Wuhan, China. Virus Res **2020**; 286:198043. Available at: https://pubmed.ncbi.nlm.nih.gov/32502551.

43. Luo L, Liu D, Liao X, et al. Modes of contact and risk of transmission in COVID-19 among close contacts. medRxiv **2020**; :2020.03.24.20042606. Available at: https://www.medrxiv.org/content/medrxiv/early/2020/03/26/2020.03.24.20042606.full.pdf.

44. Mahapure KS, Kulkarni NS. Asymptomatic Transmission of Severe Acute Respiratory Syndrome-Coronavirus 2 within a Family Cluster of 26 Cases: Why Quarantine is Important? J Glob Infect Dis **2020**; 12:115–116. Available at: https://pubmed.ncbi.nlm.nih.gov/32774003.

45. Park YJ, Choe YJ, Park O, et al. Contact Tracing during Coronavirus Disease Outbreak, South Korea, 2020. Emerg Infect Dis **2020**; 26.

46. Pollán M, Pérez-Gómez B, Pastor-Barriuso R, et al. Prevalence of SARS-CoV-2 in Spain (ENE-COVID): a nationwide, population-based seroepidemiological study. Lancet **2020**; 396:535–544.

47. Pung R, Chiew CJ, Young BE, et al. Investigation of three clusters of COVID-19 in Singapore: implications for surveillance and response measures. Lancet **2020**; 395:1039–1046.

48. Rosenberg ES, Dufort EM, Blog DS, et al. COVID-19 Testing, Epidemic Features, Hospital Outcomes, and Household Prevalence, New York State-March 2020. Clin Infect Dis **2020**; :ciaa549. Available at: https://pubmed.ncbi.nlm.nih.gov/32382743.

49. Sun WW, Ling F, Pan JR, et al. [Epidemiological characteristics of 2019 novel coronavirus family clustering in Zhejiang Province]. Zhonghua Yu Fang Yi Xue Za Zhi **2020**; 54:E027.

50. van der Hoek W, Backer JA, Bodewes R, et al. [The role of children in the transmission of SARS-CoV-2]. Ned Tijdschr Geneeskd **2020**; 164.

51. Wang X, Zhou Q, He Y, et al. Nosocomial outbreak of COVID-19 pneumonia in Wuhan, China. Eur Respir J **2020**; 55:2000544. Available at: https://pubmed.ncbi.nlm.nih.gov/32366488.

52. Wang Y, Tian H, Zhang L, et al. Reduction of secondary transmission of SARS-CoV-2 in households by face mask use, disinfection and social distancing: a cohort study in Beijing, China. BMJ Glob Heal **2020**; 5:e002794. Available at: https://pubmed.ncbi.nlm.nih.gov/32467353.

53. Wang Z, Ma W, Zheng X, Wu G, Zhang R. Household transmission of SARS-CoV-2. J Infect **2020**; 81:179–182. Available at: https://pubmed.ncbi.nlm.nih.gov/32283139.

54. Wang X, Pan Y, Zhang D, et al. Basic epidemiological parameter values from data of real-world in mega-cities: the characteristics of COVID-19 in Beijing, China. **2020**;

55. Wu J, Huang Y, Tu C, et al. Household Transmission of SARS-CoV-2, Zhuhai, China, 2020. Clin Infect Dis **2020**; Available at: https://doi.org/10.1093/cid/ciaa557.

56. Wu Y, Song S, Kao Q, Kong Q, Sun Z, Wang B. Risk of SARS-CoV-2 infection among contacts of individuals with COVID-19 in Hangzhou, China. Public Health **2020**; 185:57–59. Available at: http://www.sciencedirect.com/science/article/pii/S0033350620301785.

57. Xin H, Jiang F, Xue A, et al. Risk factors associated with occurrence of COVID‐19 among household persons exposed to patients with confirmed COVID‐19 in Qingdao Municipal, China. Transbound Emerg Dis **2020**;

58. Yousaf AR, Duca LM, Chu V, et al. A prospective cohort study in non-hospitalized household contacts with SARS-CoV-2 infection: symptom profiles and symptom change over time. Clin Infect Dis **2020**;

59. Yu H-J, Hu Y-F, Liu X-X, et al. Household infection: The predominant risk factor for close contacts of patients with COVID-19. Travel Med Infect Dis **2020**; 36:101809. Available at: https://pubmed.ncbi.nlm.nih.gov/32592904.

60. Yung CF, Kam K-Q, Chong CY, et al. Household Transmission of Severe Acute Respiratory Syndrome Coronavirus 2 from Adults to Children. J Pediatr **2020**; 225:249–251.

61. Zhang JZ, Zhou P, Han DB, et al. [Investigation on a cluster epidemic of COVID-19 in a supermarket in Liaocheng, Shandong province]. Zhonghua Liu Xing Bing Xue Za Zhi **2020**; 41:E055. Available at: http://europepmc.org/abstract/MED/32340093.

62. Zhang W, Cheng W, Luo L, et al. Secondary Transmission of Coronavirus Disease from Presymptomatic Persons, China. Emerg Infect Dis **2020**; 26:1924–1926.

63. Zhang J, Litvinova M, Liang Y, et al. Changes in contact patterns shape the dynamics of the COVID-19 outbreak in China. Science (80- ) **2020**; 368:1481–1486. Available at: https://science.sciencemag.org/content/sci/368/6498/1481.full.pdf.

64. Chu V, Freeman-Ponder B, Lindquist S, et al. Investigation and Serologic Follow-Up of Contacts of an Early Confirmed Case-Patient with COVID-19, Washington, USA. Emerg Infect Dis J **2020**; 26:1671. Available at: https://wwwnc.cdc.gov/eid/article/26/8/20-1423_article.

65. Hijnen D, Marzano AV, Eyerich K, et al. SARS-CoV-2 Transmission from Presymptomatic Meeting Attendee, Germany. Emerg Infect Dis J **2020**; 26:1935. Available at: https://wwwnc.cdc.gov/eid/article/26/8/20-1235_article.

66. Park SY, Kim YM, Yi S, et al. Coronavirus Disease Outbreak in Call Center, South Korea. Emerg Infect Dis **2020**; 26:1666–1670.

67. Baker MA, Rhee C, Fiumara K, et al. COVID-19 infections among HCWs exposed to a patient with a delayed diagnosis of COVID-19. Infect Control Hosp Epidemiol **2020**; :1–2. Available at: https://pubmed.ncbi.nlm.nih.gov/32456720.

68. Canova V, Lederer Schläpfer H, Piso RJ, et al. Transmission risk of SARS-CoV-2 to healthcare workers -observational results of a primary care hospital contact tracing. Swiss Med Wkly **2020**; 150:w20257.

69. Chen Y, Tong X, Wang J, et al. High SARS-CoV-2 antibody prevalence among healthcare workers exposed to COVID-19 patients. J Infect **2020**; 81:420–426. Available at: https://pubmed.ncbi.nlm.nih.gov/32504745.

70. Gao M, Yang L, Chen X, et al. A study on infectivity of asymptomatic SARS-CoV-2 carriers. Respir Med **2020**; 169:106026. Available at: https://pubmed.ncbi.nlm.nih.gov/32513410.

71. Ghinai I, McPherson TD, Hunter JC, et al. First known person-to-person transmission of severe acute respiratory syndrome coronavirus 2 (SARS-CoV-2) in the USA. Lancet (London, England) **2020**; 395:1137–1144. Available at: http://europepmc.org/abstract/MED/32178768.

72. Hara T, Yamamoto C, Sawada R, et al. Infection risk in a gastroenterological ward during a nosocomial COVID-19 infection event. J Med Virol **2020**; :10.1002/jmv.25853. Available at: https://pubmed.ncbi.nlm.nih.gov/32320062.

73. Heinzerling A, Stuckey MJ, Scheuer T, et al. Transmission of COVID-19 to Health Care Personnel During Exposures to a Hospitalized Patient - Solano County, California, February 2020. MMWR Morb Mortal Wkly Rep **2020**; 69:472–476.

74. COVID-19 National Emergency Response Center Korea Centers for Disease Control and Prevention, E and CMT. Coronavirus Disease-19: Summary of 2,370 Contact Investigations of the First 30 Cases in the Republic of Korea. Osong Public Heal Res Perspect **2020**; 11:81–84.

75. Liu T, Liang W, Zhong H, et al. Risk factors associated with COVID-19 infection: a retrospective cohort study based on contacts tracing. Emerg Microbes Infect **2020**; 9:1546–1553. Available at: https://doi.org/10.1080/22221751.2020.1787799.

76. Lombardi A, Consonni D, Carugno M, et al. Characteristics of 1,573 healthcare workers who underwent nasopharyngeal swab for SARS-CoV-2 in Milano, Lombardy, Italy. Clin Microbiol Infect **2020**;

77. Ng K, Poon BH, Kiat Puar TH, et al. COVID-19 and the Risk to Health Care Workers: A Case Report. Ann Intern Med **2020**; 172:766–767.

78. Pini R, Faggioli G, Vacirca A, et al. Is it Possible to Safely Maintain a Regular Vascular Practice During the COVID-19 Pandemic? Eur J Vasc Endovasc Surg **2020**; 60:127–134. Available at: https://pubmed.ncbi.nlm.nih.gov/32499169.

79. Saban O, Levy J, Chowers I. Risk of SARS-CoV-2 transmission to medical staff and patients from an exposure to a COVID-19-positive ophthalmologist. Graefes Arch Clin Exp Ophthalmol **2020**; :1–4. Available at: https://pubmed.ncbi.nlm.nih.gov/32567041.

80. Schneider KN, Correa-Martínez CL, Gosheger G, et al. Assessing the spreading potential of an undetected case of COVID-19 in orthopaedic surgery. Arch Orthop Trauma Surg **2020**; :1–7. Available at: https://pubmed.ncbi.nlm.nih.gov/32524227.

81. Singh G, Srinivas G, Jyothi EK, Gayatri LK, Gaitonde R, Soman B. Containing the first outbreak of COVID-19 in a healthcare setting in India: The sree chitra experience. Indian J Public Heal **2020**; 64:S240-s242.

82. Wee LE, Hsieh JYC, Phua GC, et al. Respiratory surveillance wards as a strategy to reduce nosocomial transmission of COVID-19 through early detection: The experience of a tertiary-care hospital in Singapore. Infect Control Hosp Epidemiol **2020**; :1–6. Available at: https://pubmed.ncbi.nlm.nih.gov/32381147.

83. Wee LEI, Sim XYJ, Conceicao EP, et al. Containing COVID-19 outside the isolation ward: The impact of an infection control bundle on environmental contamination and transmission in a cohorted general ward. Am J Infect Control **2020**; :S0196-6553(20)30569–1. Available at: https://pubmed.ncbi.nlm.nih.gov/32599101.

84. Wee LE, Sim XYJ, Conceicao EP, et al. Containment of COVID-19 cases among healthcare workers: The role of surveillance, early detection, and outbreak management. Infect Control Hosp Epidemiol **2020**; :1–7.

85. Wendt R, Nagel S, Nickel O, et al. Comprehensive investigation of an in-hospital transmission cluster of a symptomatic SARS-CoV-2-positive physician among patients and healthcare workers in Germany. Infect Control Hosp Epidemiol **2020**; :1–3. Available at: https://pubmed.ncbi.nlm.nih.gov/32489162.

86. Wong SCY, Kwong RT, Wu TC, et al. Risk of nosocomial transmission of coronavirus disease 2019: an experience in a general ward setting in Hong Kong. J Hosp Infect **2020**; 105:119–127.

87. Hamner L, Dubbel P, Capron I, et al. High SARS-CoV-2 Attack Rate Following Exposure at a Choir Practice — Skagit County, Washington, March 2020. 2020.

88. James A, Eagle L, Phillips C, et al. High COVID-19 Attack Rate Among Attendees at Events at a Church - Arkansas, March 2020. MMWR Morb Mortal Wkly Rep **2020**; 69:632–635.

89. Aghaali M, Kolifarhood G, Nikbakht R, Saadati HM, Hashemi Nazari SS. Estimation of the serial interval and basic reproduction number of COVID-19 in Qom, Iran, and three other countries: A data-driven analysis in the early phase of the outbreak. Transbound Emerg Dis **2020**; :10.1111/tbed.13656. Available at: https://pubmed.ncbi.nlm.nih.gov/32473049.

90. Barrett PM, Bambury N, Kelly L, Condon R, Crompton J, Sheahan A. Measuring the effectiveness of an automated text messaging active surveillance system for COVID-19 in the south of Ireland, March to April 2020. Euro Surveill **2020**; 25.

91. Ge R, Tian M, Gu Q, et al. The role of close contacts tracking management in COVID-19 prevention: A cluster investigation in Jiaxing, China. J Infect **2020**; 81:e71–e74. Available at: https://pubmed.ncbi.nlm.nih.gov/32268181.

92. Han X, Wei X, Alwalid O, et al. Severe Acute Respiratory Syndrome Coronavirus 2 among Asymptomatic Workers Screened for Work Resumption, China. Emerg Infect Dis **2020**; 26.

93. Hong L-X, Lin A, He Z-B, et al. Mask wearing in pre-symptomatic patients prevents SARS-CoV-2 transmission: An epidemiological analysis. Travel Med Infect Dis **2020**; 36:101803. Available at: http://www.sciencedirect.com/science/article/pii/S1477893920302994.

94. Jia J, Hu X, Yang F, et al. Epidemiological Characteristics on the Clustering Nature of COVID-19 in Qingdao City, 2020: A Descriptive Analysis. Disaster Med Public Health Prep **2020**; :1–5. Available at: https://pubmed.ncbi.nlm.nih.gov/32228732.

95. Kwok KO, Wong VWY, Wei WI, Wong SYS, Tang JW-T. Epidemiological characteristics of the first 53 laboratory-confirmed cases of COVID-19 epidemic in Hong Kong, 13 February 2020. Eurosurveillance **2020**; 25:2000155. Available at: https://www.eurosurveillance.org/content/10.2807/1560-7917.ES.2020.25.16.2000155.

96. Liu Z, Chu R, Gong L, Su B, Wu J. The assessment of transmission efficiency and latent infection period on asymptomatic carriers of SARS-CoV-2 infection. Int J Infect Dis **2020**; Available at: http://www.sciencedirect.com/science/article/pii/S1201971220304719.

97. Liu L, Lei X, Xiao X, et al. Epidemiological and Clinical Characteristics of Patients With Coronavirus Disease-2019 in Shiyan City, China. Front Cell Infect Microbiol **2020**; 10:284. Available at: https://pubmed.ncbi.nlm.nih.gov/32574282.

98. Valent F, Gallo T, Mazzolini E, et al. A cluster of COVID-19 cases in a small Italian town: a successful example of contact tracing and swab collection. Clin Microbiol Infect **2020**; 26:1112–1114. Available at: https://pubmed.ncbi.nlm.nih.gov/32344169.

99. Wong J, Koh WC, Momin RN, Alikhan MF, Fadillah N, Naing L. Probable causes and risk factors for positive SARS-CoV-2 test in recovered patients: Evidence from Brunei Darussalam. medRxiv **2020**; :2020.04.30.20086082. Available at: https://www.medrxiv.org/content/medrxiv/early/2020/05/05/2020.04.30.20086082.full.pdf.

100. Zhang HJ, Su YY, Xu SL, et al. Asymptomatic and symptomatic SARS-CoV-2 infections in close contacts of COVID-19 patients: a seroepidemiological study. Clin Infect Dis **2020**;
